# Gene therapy for genetic diseases: challenges and future directions

**DOI:** 10.1002/mco2.70091

**Published:** 2025-02-13

**Authors:** Beibei Qie, Jianghua Tuo, Feilong Chen, Haili Ding, Lei Lyu

**Affiliations:** ^1^ Institute of Sports Medicine and Health, School of Sports Medicine and Health Chengdu Sport University Chengdu China

**Keywords:** gene editing, gene replacement, gene therapy, muscular disorders, rare genetic diseases

## Abstract

Genetic diseases constitute the majority of rare human diseases, resulting from abnormalities in an individual's genetic composition. Traditional treatments offer limited relief for these challenging conditions. In contrast, the rapid advancement of gene therapy presents significant advantages by directly addressing the underlying causes of genetic diseases, thereby providing the potential for precision treatment and the possibility of curing these disorders. This review aims to delineate the mechanisms and outcomes of current gene therapy approaches in clinical applications across various genetic diseases affecting different body systems. Additionally, genetic muscular disorders will be examined as a case study to investigate innovative strategies of novel therapeutic approaches, including gene replacement, gene suppression, gene supplementation, and gene editing, along with their associated advantages and limitations at both clinical and preclinical levels. Finally, this review emphasizes the existing challenges of gene therapy, such as vector packaging limitations, immunotoxicity, therapy specificity, and the subcellular localization and immunogenicity of therapeutic cargos, while discussing potential optimization directions for future research. Achieving delivery specificity, as well as long‐term effectiveness and safety, will be crucial for the future development of gene therapies targeting genetic diseases.

## Graphical abstract

The graphical abstract provides an overview of gene therapy approaches, detailing the components of the therapy and the various delivery routes. Both in vivo and ex vivo strategies facilitate the implementation of gene replacement, gene suppression, gene supplementation, and gene editing. Therapeutic effects have been demonstrated across a spectrum of genetic diseases as well as in various specific degenerative diseases.

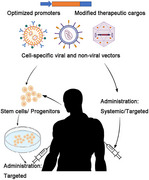

## 1 INTRODUCTION

Genetic diseases account for approximately 80% of all rare human diseases, affecting about one in 17 individuals, given the sheer number of several thousand such disorders.^1^ With the rapid accumulation of human genetic sequence data and advancements in molecular genetics research over the past two decades, the mechanisms underlying many genetic diseases have become better understood.^1^, ^2^, ^3^ However, traditional symptomatic treatments for these conditions often provide only limited relief from clinical symptoms. There is an urgent need to develop effective therapies that target the root causes of these disorders. Genetic diseases arise from alterations in the genome, which may include mutations, deletions, or insertions in DNA sequences. Consequently, gene therapy, which can specifically target pathogenic genetic materials and deliver a long‐lasting therapeutic effect, represents a revolutionary approach to treating genetic diseases by directly addressing the genetic defects at their source.

In the last century, advancements in the transformation and recombination of human genomic DNA,^4^ alongside viral‐based delivery of exogenous genetic material and insights into virus‐mediated therapy‐related immunity and genotoxicity, have facilitated the gradual development of gene therapy amidst various challenges.^5^, ^6^, ^7^ The first application of retroviral‐based gene delivery occurred in 1990 for a patient with adenosine deaminase‐severe combined immunodeficiency.^8^ Since the beginning of this century, rapid progress in research on viral vectors—such as adenoviruses, adeno‐associated viruses (AAV), and lentiviruses—as well as nonviral vectors has led to a swift transition of gene therapy from theoretical research to clinical applications.^9^ Notably, the effectiveness of gene therapy has been reported in patients with Leber's congenital amaurosis (LCA) and Hemophilia B during the first decade of the 2000s.^10^, ^11^ The first AAV‐based gene therapy was approved for reverse lipoprotein lipase deficiency in Europe in 2012.^12^ Over the past decade, the rapid maturation of gene delivery technology and therapeutic strategies, along with clinical response strategies for managing adverse reactions to gene therapy, has resulted in the development of diverse treatment approaches, including gene replacement, gene suppression, gene supplementation, and gene editing for both basic and clinical studies of multiple genetic diseases.^13^, ^14^, ^15^ Currently, there are approximately 500 registered clinical trials for monogenic diseases, with 11 gene therapy medicines approved by the United States Food and Drug Administration (US FDA) since 2017, including seven approvals occurring after 2023.^16^

Given the rapid advancements in gene therapy research and clinical trials in recent years, it is essential to summarize and update the current status of applications, existing challenges, and future prospects of these approaches. This review aims to address these needs by summarizing several key aspects of gene therapy. First, the types and mechanisms of currently employed therapeutic approaches, as well as the delivery systems for gene therapy, will be briefly outlined to construct an overview of the field. Next, the application of gene therapy in genetic diseases will be discussed, beginning with the mechanisms and outcomes of US FDA‐approved medications. Acknowledging the impossibility of covering all gene therapy‐targeted diseases in a single review, this discussion will focus on representative details of preclinical studies and clinical trial progress in genetic muscular diseases. This focus is warranted as the successful treatment of muscle diseases requires addressing numerous shared therapeutic challenges, such as delivery efficiency and specificity, due to the high vector dosage required by the substantial muscle mass,^17^ the specificities among different muscle tissues,^18^ and the central nervous system (CNS) symptoms present in certain muscle diseases.^19^, ^20^ The review will emphasize how novel gene therapy approaches, including gene replacement and gene editing, have the potential to transform the treatment paradigm for genetic disorders. Furthermore, the major challenges currently facing gene therapy in these disorders will be discussed, including packaging limitations, immunotoxicity, and delivery/expression specificity, along with potential solutions. The advancement of gene therapy techniques represents a significant opportunity to usher in an era of personalized, targeted medicine that can dramatically improve cure rates and quality of life for individuals with genetic disorders worldwide. Enhancing the specificity, effectiveness, and safety of both vectors and cargos will be crucial for the future success of gene therapy in these diseases.

## 2 OVERVIEW OF GENE THERAPY IN GENETIC DISEASES

### 2.1 Gene therapy approaches

**FIGURE 1:**
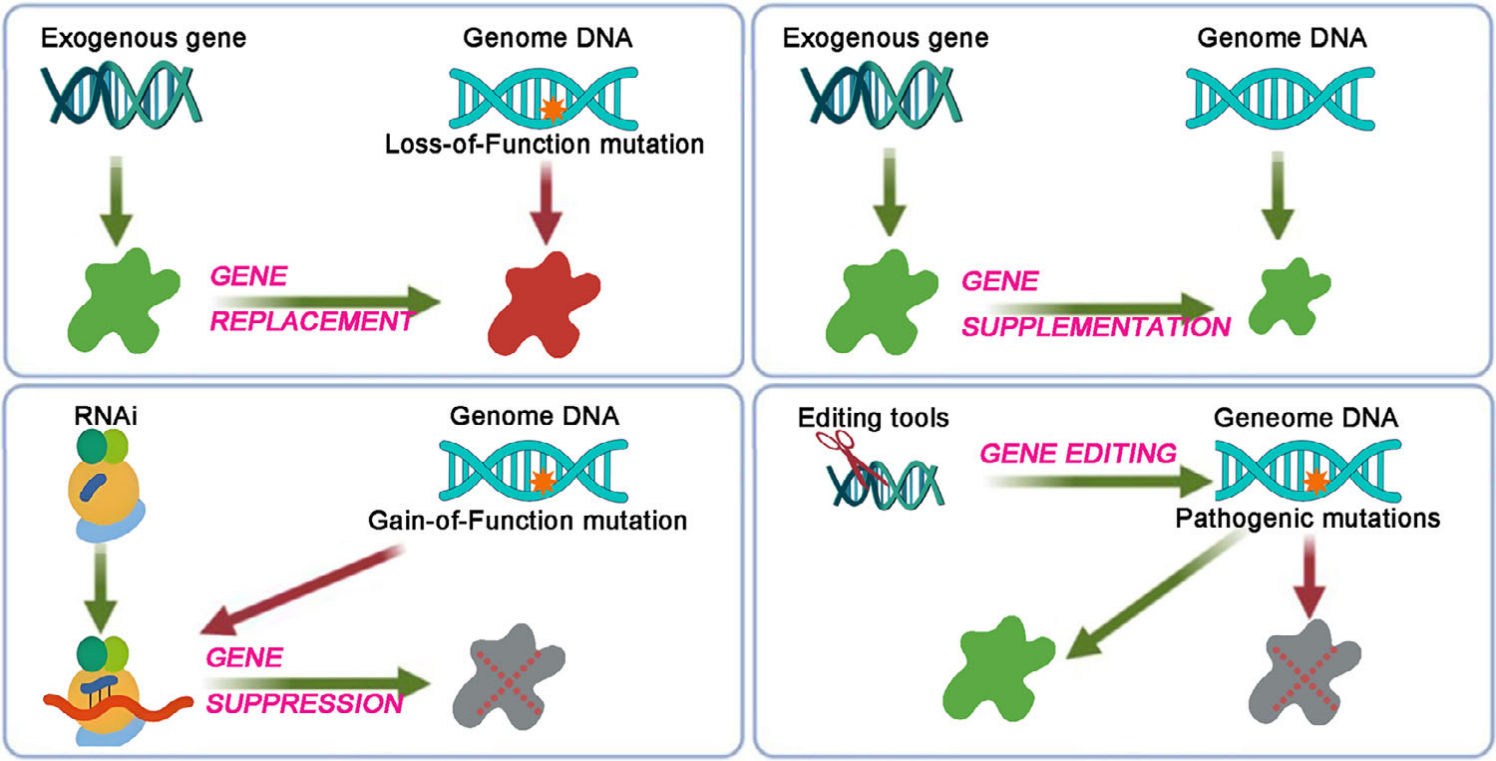
Gene therapy strategies for genetic diseases. This schematic diagram illustrates the treatment processes associated with various gene therapy strategies, including gene replacement, gene supplementation, gene suppression, and gene editing. Green solid patterns represent functional products, while red solid patterns denote pathogenic products. Gray solid patterns with a red fork indicate inhibited products.

Over the last few decades, numerous new treatment options have been researched and developed, transitioning from bench to bedside. Among these, gene therapies have emerged that can persistently adjust RNA transcription levels or directly target the DNA sequences of pathogenic genes to modify gene product expression.^21^ These approaches—gene replacement, gene suppression, gene supplementation, and gene editing (Figure 1)—aim to correct gene mutations of various profiles with limited or even single treatments.^22^, ^23^, ^24^, ^25^, ^26^ This significantly expands the scope of gene therapy for different types of genetic disorders, restoring the expression of normal genes to not only modify but potentially cure the diseases.

#### 2.1.1 Gene replacement

Among the aforementioned approaches, gene replacement currently stands out as the most clinically validated method for addressing genetic disorders, having been employed in 10 of the 11 US FDA‐approved medicines (Table 1). The primary objective of this approach is to deliver either a full or a truncated yet functional version of an exogenous gene into the target cells of the patients. This delivery aims to introduce a functional copy of the identified mutated gene, thereby restoring normal gene function. However, several limitations exist regarding this approach. The capacity for packaging the functional exogenous gene is constrained by the delivery systems,^27^ while gain‐of‐function and polygenic mutations are generally unsuitable for this therapy.

**TABLE 1:** Summary of US FDA approved gene therapy approaches.

Disease	Therapy	Method/vector	Promoter/transgene	Delivery method/recommend dosage	Age range	Primary outcomes	Adverse event	Approved year
Leber congenital amaurosis	LUXTURNA (voretigene neparvovec‐rzyl)	Gene replacement/rAAV2	CAG promoter/human RPE65 protein	Subretinal injection, 1.5 × 10^11^ vg/kg for each eye	Adult and pediatric	MLMT score change for bilateral eyes, (treated vs. control, median (min, max)): 2 (0, 4) vs. 0 (−1, 2), *p* = 0.001; MLMT score change for first‐treated eye: 2 (0, 4) vs. 0 (−1, 1), *p* = 0.003	Conjunctival hyperemia, cataract, increased intraocular pressure, retinal tear, dellen, macular hole, subretinal deposits, eye inflammation, eye irritation, eye pain, and maculopathy	2017^63^, ^64^, ^65^
β‐Thalassemia	ZYNTEGLO (betibeglogene autotemcel)	Gene replacement/BB305 LV	Erythroid lineage cells specific promoter/s β^A‐T87Q^‐globin	Intravenous infusion, 5 × 10^6^ CD34+ cells/kg	Adult and pediatric	Transfusion Independence: 32/36 (89%) (95% CI: 74, 97); weighted average total Hb during TI (g/dL): 11.5 (95% CI: 9.3, 13.7)	Nonlaboratory events: mucositis, febrile neutropenia, vomiting, pyrexia (fever), alopecia (hair loss), epistaxis (nose bleed), abdominal pain, musculoskeletal pain, cough, headache, diarrhea, rash, constipation, nausea, decreased appetite, pigmentation disorder, and pruritus; Laboratory abnormalities: neutropenia, thrombocytopenia, leukopenia, anemia, and lymphopenia	2022^73^, ^74^, ^75^
Sickle cell disease	LYFGENIA (lovotibeglogene autotemcel)	Gene replacement/BB305 LV	Erythroid lineage cells specific promoter/s β^A‐T87Q^‐globin	Intravenous infusion, 3 × 10^6^ CD34+ cells/kg	≥12 years	VOE‐CR: 28/32 (88%) (95% CI: 71, 97); sVOE‐CR: 30/32 (94%) (95% CI: 79, 99)	Stomatitis, thrombocytopenia, neutropenia, febrile neutropenia, anemia, and leukopenia	2023^72^, ^77^
Sickle cell disease (SCD) and transfusion‐dependent ß‐thalassemia (TDT)	CASGEVY (exagamglogene autotemcel)	Gene editing/gene edited autologous CD34+ HSCs	Cas9 and sgRNA targeting erythroid‐specific enhancer region of BCL11A	Intravenous infusion, 3 × 10^6^ CD34+ cells/kg	≥12 years	VF12 response rate: 29/31 (93.5%, 98% one‐sided CI: 77.9%, 100.0%); HF12 response rate:30/30 s (100%, 98% one‐sided CI: 87.8%, 100.0%)	Laboratory abnormalities: neutropenia, thrombocytopenia, leukopenia, anemia, and lymphopenia; Nonlaboratory events: mucositis and febrile neutropenia in SCD and TDT, and decreased appetite in SCD	2023^82^, ^83^, ^84^
Hemophilia B	HEMGENIX (etranacogene dezaparvovec‐drlb)	Gene replacement/rAAV5	Hybrid human liver‐specific promoter/hFIX‐R338L	Single IV infusion, 2 × 10^13^ vg/kg	Adult	Mean ABR (bleeds/year) (months 7–18 posttreatment vs. lead‐in): 1.9 (95% CI: 1.0, 3.4) vs. 4.1 (95% CI: 3.2, 5.4); ABR ratio: 0.46 (95% CI: 0.26, 0.81)	Elevated ALT, headache, blood creatine kinase elevations, flu‐like symptoms, infusion‐related reactions, fatigue, malaise, and elevated AST	2022^91^, ^92^, ^96^
Hemophilia A	ROCTAVIAN (valoctocogene roxaparvovec‐rvox)	Gene replacement/rAAV5	Hybrid human liver‐specific promoter/hFVIII‐SQ	Single IV infusion, 6 × 10^13^ vg/kg	Adult	Mean (SD) ABR (bleeds/year) (posttreatment vs. lead‐in): 2.6 (6.2) vs. 5.4 (6.9), mean difference: −2.8 (95% CI: −4.3, −1.2)	Nonlaboratory events: nausea, fatigue, headache, infusion‐related reactions, vomiting, and abdominal pain; laboratory events: e ALT, AST, LDH, CPK, factor VIII activity levels, GGT, and bilirubin > ULN	2023^94^, ^95^
Hemophilia B	BEQVEZ (Fidanacogene elaparvovec‐dzkt)	Gene replacement/rAAVRh74var	Liver‐specific human α1‐antitrypsin promoter/hFIX‐R338L	Single IV infusion, 5 × 10^11^ vg/kg	Adult	Model derived mean ABR [bleeds/year] (95% CI, post‐BEQVEZ vs. baseline): 2.5 (1.0, 3.9) vs. 4.5 (1.9, 7.2) mean difference: −2.1 (95% CI: −4.8, 0.7)	Elevated transaminases	2024^90^
Dystrophic epidermolysis bullosa	VYJUVEK (beremagene geperpavec)	Gene replacement/HSV‐1	COL7A1	External application of wound with excipient gel	≥0.5 years	Wound closure assessment (treatment vs. placebo): around weeks 24: 20 (65%) vs. 8 (26%), treatment difference 39% (95% CI: 14, 63, *p* = 0.012); around weeks 10: 21 (68%) vs. 7 (23%), treatment difference 45% (95% CI: 22, 69, *p* = 0.003)	Itching, chills, redness, rash, cough, and runny nose	2023^100^, ^101^
Spinal muscular atrophy (SMA)	ZOLGENSMA (onasemnogene abeparvovec‐xioi)	Gene replacement/rAAV9	CAG promoter/human SMN	Single IV infusion, 1.1 × 10^14^ vg/kg	<2 years	9 (75.0%) patients were able to sit without support for ≥30 s, 2 (16.7%) were able to stand and walk without assistance 24 weeks after administration in high dosage group (at least 1.1 × 10^14^ vg/kg)	Elevated aminotransferases and vomiting; cases of acute liver failure with fatal outcomes was reported	2019^106^, ^107^, ^109^, ^111^, ^112^
Aromatic L‐amino acid decarboxylase (AADC) deficiency	KEBILIDI (eladocagene exuparvovec‐tneq)	Gene replacement/rAAV2	CMV promoter/human DDC gene	Single intraputaminal infusion, 1.8 × 10^11^ vg/kg	Adult and pediatric	Gross motor milestone achievement at week 48: 8/12 (67%) in treated group and 0/43 in untreated group	Dyskinesia, pyrexia, hypotension, anemia, salivary hypersecretion, hypokalemia, hypophosphatemia, insomnia, hypomagnesemia, and procedural complications	2024^119^, ^136^
Duchenne muscular dystrophy (DMD)	ELEVIDYS (delandistrogene moxeparvovec‐rokl)	Gene replacement/rAAVrh74	MHCK7/microdystrophin includes spectrin repeats 1–3 and 24, as well as hinges 1, 2, and 4	Single IV infusion, 1.33 × 10^14^ vg/kg	≥4 years	NSAA total score: (*p* = 0.37); mean microdystrophin expression at week 12: 34.3% (*N* = 17, SD: 41.0%)	Vomiting and nausea, liver injury, pyrexia, and thrombocytopenia	2024^130^, ^131^, ^132^, ^133^, ^134^

#### 2.1.2 Gene supplementation

The approach shares many characteristics and limitations with gene replacement; however, it does not target an endogenous mutation. Instead, these exogenous cargos are designed to supplement under‐expressed endogenous genes, thereby addressing the pathological features of various diseases, including metabolic disorders, aging, and infections.^28^, ^29^, ^30^, ^31^ The therapeutic efficacy of gene supplementation delivering endocrine proteins, such as growth factors and insulin, has been studied in animal models of obesity and diabetes.^29^ Additionally, the supplementation of recombinant neutralizing antibodies (NAbs) in this approach has been explored in the treatment of HIV‐1 infection.^31^

#### 2.1.3 Gene suppression

Combining RNA interference (RNAi) technology with in vivo delivery methods, gene suppression is primarily applied in monogenic diseases characterized by gain‐of‐function mutations.^32^ However, challenges related to maintaining RNA stability, as well as frequently reported safety concerns such as toxicity and off‐target effects, must be carefully considered prior to the clinical application of this approach.^33^

#### 2.1.4 Gene editing

Another promising therapeutic strategy is gene editing, which has already been employed in clinical trials for various rare diseases.^34^ Gene editing platforms are versatile tools that facilitate the generation of specific, site‐directed DNA insertions, deletions, and substitutions, rendering them suitable for disease treatment and trait modification since the discovery of DNA's double‐helix structure.^34^ The development of zinc‐finger nucleases, transcription activator‐like effector nucleases, and Clustered Regularly Interspaced Short Palindromic Repeats (CRISPR)/CRISPR‐associated (Cas) systems has enabled precise and efficient editing of DNA within the nuclear genome.^15^ Among these, the CRISPR/Cas system can be easily programmed using a single guide RNA (sgRNA) to target nearly any sgRNA‐complementary sequence that is followed by a protospacer‐adjacent motif (PAM), thereby guiding the Cas protein to achieve the desired editing function, which highlights its utility and accuracy in performing targeted genome editing.^35^ Moreover, modifications to either Cas proteins or sgRNAs have significantly enhanced the functionality of this system, including CRISPR‐based activation (CRISPRa), inhibition (CRISPRi), base editing, and prime editing, making it an extremely promising tool for gene therapy.^36^ Consequently, since the initial demonstration of programmable DNA cleavage, the CRISPR/Cas system has been investigated across a wide spectrum of human genetic diseases.^37^ Despite the presence of numerous challenges, such as off‐target editing, lack of efficient and specific delivery, immune responses to nucleases, and packaging limitations,^38^, ^39^ gene editing offers a powerful means to directly and permanently correct genomic mutations.

### 2.2 Delivery systems

Another crucial step in gene therapy is ensuring the accurate and safe delivery of the aforementioned cargos to targeted organs in vivo. Several delivery systems, which rely on either viral or nonviral vectors, are currently under investigation for the gene therapy of genetic diseases.^40^

#### 2.2.1 Viral delivery system

Due to their natural ability to infect cells with specific tropism, viruses can penetrate biological membranes and deliver target genes to their intended locations, making them the most suitable candidate vectors for gene therapy. Numerous types of viruses have been modified into vectors for gene therapy and cell therapy, based on various therapeutic principles and target tissues. These include AAV, lentivirus, retrovirus, adenovirus, herpes simplex virus (HSV), oncolytic viruses, chickenpox virus, measles virus, and Newcastle disease virus.^40^

Among the various viral delivery tools, AAV vectors are widely regarded as the most accepted option for treating rare genetic diseases. AAVs are nonenveloped, single‐stranded DNA viruses characterized by the initial attachment of glycan moieties to the cell surface across different serotypes.^41^ The packaged recombinant AAV (rAAV) vectors contain the transgene of interest along with essential regulatory elements, rather than viral genes. These vectors exhibit low toxicity and immunogenicity for systemic application, while different serotypes demonstrate distinct tissue tropisms in vivo.^42^, ^43^, ^44^, ^45^ Consequently, various serotypes of vectors have shown considerable potential for delivering genetic material into diverse tissues and are commonly employed as the delivery system in current clinical trials.^46^ Additionally, AAVs isolated from nonhuman primates, such as rAAV serotype rh74 (rAAVrh74) derived from rhesus monkeys, are also utilized due to their potentially lower immunogenicity compared with serotypes derived from humans.^47^ However, it is important to note that AAV, being a small virus, can package a linear single‐stranded DNA genome of approximately 4.7 kb, which limits its capacity to carry the full length of therapeutic genes, particularly large target genes like dystrophin.^48^ Furthermore, the presence of pre‐existing NAbs against AAV capsids in a significant portion of the human population, resulting from natural AAV infections, poses additional challenges to clinical applications.^49^

Lentiviral vectors (LVs), derived from human immunodeficiency virus and characterized by their enveloped, single‐stranded RNA structure, offer a substantial packaging capacity of up to 8 kb while maintaining low immunogenicity.^50^ These vectors are capable of transducing both dividing and nondividing mammalian cells through the reverse transcription of their RNA genome and subsequent integration into the host genome.^51^, ^52^ Gene therapies utilizing LVs are widely employed in the treatment of hematological diseases, particularly through the correction of autologous hematopoietic stem and progenitor cells (HSPCs).^53^ Furthermore, a recent study has demonstrated that LVs can be utilized for cargo delivery to muscle tissues via muscle‐specific promoters,^54^ thereby broadening the potential applications of this viral vector in the treatment of various genetic diseases.

#### 2.2.2 Nonviral delivery system

A significant challenge associated with viral‐mediated in vivo delivery is that the capsid proteins of viruses are readily targeted by the immune surveillance system. This targeting can lead to the neutralization of subsequent vector injections due to acquired immunity.^55^, ^56^ Consequently, nonviral delivery approaches present several advantages over viral systems, including enhanced safety with minimal immunogenicity, high packaging capacity and efficiency at a substantially lower cost, and improved biocompatibility. Additionally, nonviral systems can be easily modified for targeted delivery to specific tissues.^57^ Vectors such as lipid nanoparticles (LNPs), synthetic polymers, and exosome‐based systems have emerged as promising candidates for the clinical applications of gene therapies.^58^

## 3 CLINICAL APPLICATIONS OF GENE THERAPY IN GENETIC DISEASES

**FIGURE 2:**
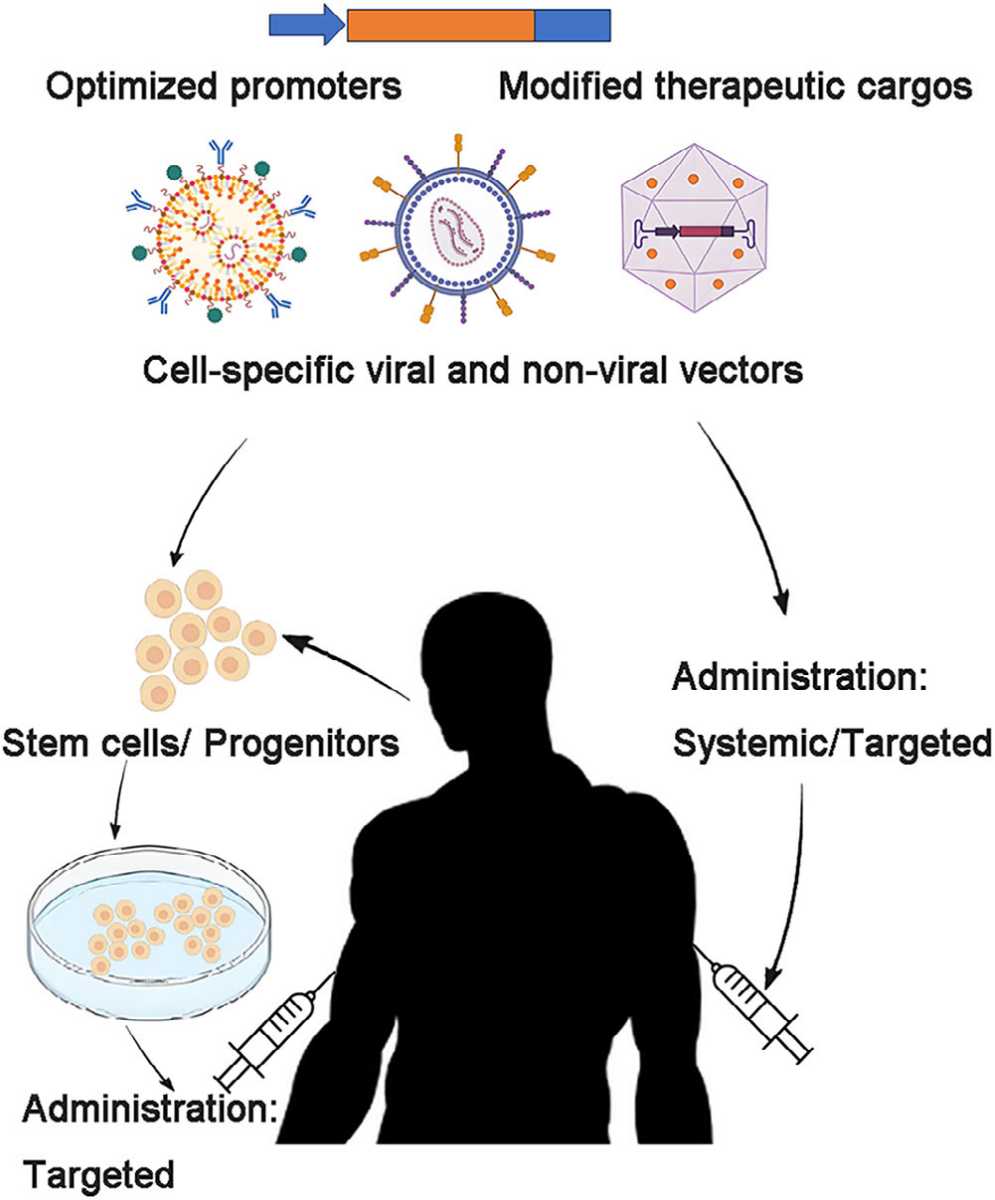
A concise overview of the main components and delivery routes for genetic diseases. An effective treatment strategy relies on the utilization of specific and efficient promoters, well‐structured cargos with accurate subcellular localization, and reliable carriers and delivery methods that target designated tissues while demonstrating low toxicity.

The successful application of gene therapy not only depends on well‐designed therapeutic approaches and delivery methods, as mentioned above, but also requires comprehensive research into the pathogenic mechanisms of diseases. This understanding is crucial for designing targeted promoters and well‐structured functional exogenous cargos, as well as for selecting appropriate delivery routes (Figure 2). Over the past decade, there has been a continuous accumulation of knowledge regarding the mechanisms underlying rare genetic diseases, leading to a proliferation of preclinical studies and clinical trials across a broad spectrum of disorders, including those affecting the visual, neurological, hematological, and muscular systems.^13^, ^14^, ^40^Among these research advancements, 11 medicines have been approved by the US FDA to date, as summarized in Table 1, with a brief discussion of their mechanisms and outcomes provided below.

### 3.1 Leber congenital amaurosis

LCA is a severe inherited retinal dystrophy that is present from birth, characterized by significant visual impairment or blindness in early infancy. More than 20 gene mutations are currently known to be associated with LCA. Research on population genetic screening for these mutations in a cohort of Danish LCA probands revealed that RPE65 was the most frequently mutated gene, with biallelic variants found in 16% of the population.^59^ RPE65, expressed in the retinal pigment epithelium (RPE), encodes an all‐trans‐retinyl ester isomerase that maintains the function of both rod and cone photoreceptors by participating in the biochemical recycling of chromophores in the visual cycle.^60^ Mutations in RPE65 lead to the accumulation of detrimental precursor molecules and the degeneration of RPE cells, resulting in a progressive loss of photoreceptor cells.^61^

LUXTURNA (voretigene neparvovec‐rzyl) was the first gene therapy medicine approved by the US FDA for rare genetic diseases, providing meaningful improvements in visual function for patients who previously had limited options.^16^ This therapy consists of human RPE65 cDNA with a modified Kozak sequence, driven by a cytomegalovirus (CMV) immediate early enhancer/chicken β actin (CAG) promoter, which enhances the efficiency of expression profiling and transcription. It is delivered via an AAV2 vector through subretinal surgery.^62^, ^63^ The mean bilateral multiluminance mobility testing (MLMT) score after 1 year for patients who received the therapy was significantly higher than that of the control group, with a difference of 1.6 (95% CI: 0.72–2.41, *p* = 0.0013).^64^ Results from a 3‐year follow‐up of delayed intervention (DI) patients and a 4‐year follow‐up of original intervention (OI) patients in the Phase III trial (NCT00999609) indicated mean bilateral MLMT score changes of 2.4 and 1.7, respectively, with 71% of patients passing the MLMT at the lowest light level (1 lux) by year 3. Improvements in light sensitivity and visual field were also observed in both DI and OI groups, suggesting that the benefits of the therapy persist for up to 4 years.^65^ The serious adverse events that occurred were related to the administration procedure rather than the gene therapy itself, and no harmful immune responses associated with either the viral capsid or the delivered gene were observed.^65^ Nonetheless, as the first US FDA‐approved gene therapy for rare diseases, LUXTURNA has been subject to ongoing controversy, much of which is common to other approved therapies. This includes concerns regarding the lack of full restoration of normal vision, insufficient long‐term efficacy data, and significant barriers to accessibility and broader implications due to the high personal and societal costs, with a price tag of up to 425,000 US dollars per eye.^66^

### 3.2 Hematological diseases

Hematological disorders, such as hemophilia, sickle cell disease (SCD), and thalassemia, impose a significant disease burden, surpassing that of rare diseases affecting other solid organs. This situation underscores the urgent need for new treatment options. In comparison with other cell types, hematopoietic stem cells (HSCs) are relatively straightforward to collect, culture, expand ex vivo, manipulate, and reinfuse, making them particularly suitable for gene therapy.^67^ Furthermore, systemic treatment is inherently necessary for hematological diseases. Consequently, more than half of the medications on the approved list are directed toward genetic diseases of the hematological system.

#### 3.2.1 SCD and β‐thalassemia

Both SCD and β‐thalassemia are characterized by red blood cell dysfunction resulting from mutations in the hemoglobin β subunit gene (HBB). In β‐thalassemia, there is a reduction or absence of β‐globin synthesis, while SCD is associated with a specific point mutation in HBB (Glu6Val), leading to either ineffective erythropoiesis or erythrocyte deformation.^68^, ^69^ Chronic red cell transfusion support and symptom management represent common clinical treatment strategies, whereas allogeneic HSC transplantation provides a potential cure for both diseases. However, this approach is limited by the availability of leukocyte antigen‐matched donors.^70^

Gene therapy that provides functional copies of HBB has shown effectiveness in treating these two disorders. An erythroid lineage cell‐specific promoter‐driven exogenous gene encoding adult hemoglobin (HbA) with a T87Q substitution (HbA ^T87Q^) was designed to quantitatively express therapeutic globin while inhibiting the polymerization of sickle hemoglobin.^71^, ^72^, ^73^ The therapeutic cargo was then delivered ex vivo via a LV to CD34+ cells from patients, which were subsequently reinfused following myeloablative busulfan conditioning. Therapeutic efficacy was demonstrated in both transfusion‐dependent β‐thalassemia (TDT) and SCD patients.

In the TDT groups, the goal of suspending red blood cell transfusions was achieved in most patients, regardless of whether they had non‐β^0^/β^0^ or β^0^/β^0^ genotypes, with a significant increase in total hemoglobin and HbA ^T87Q^ levels.^73^, ^74^, ^75^ Despite the absence of serious adverse events related to the therapy in both groups, a real‐world study with over 24 months of monitoring revealed that posttreatment platelet refractoriness could be induced by human leukocyte antigen antibodies stemming from the transfusion history of nonleukocyte‐depleted blood products. Additionally, endocrine dysfunction was identified as a long‐term adverse effect of busulfan. Furthermore, one patient developed polycythemia of unknown etiology, highlighting new challenges and considerations to be aware of during treatment.^76^

Similar approaches have also been studied in patients with SCD, revealing a long‐lasting expression of antisickling β‐globin and an elevation in total hemoglobin levels.^72^, ^77^ Severe vaso‐occlusive events (sVOE) were observed to resolve in all patients who experienced at least four pathological events in the 24 months prior to treatment, with a predicted increase of 23.84 years in average survival time following treatment.^77^, ^78^ Adverse events were also comparable to those associated with conventional bone marrow transplantation, with the exception of a case report detailing the development of acute myeloid leukemia (AML) in a woman 5.5 years after gene replacement therapy. Although vector insertion was detected, investigations indicated that it was unlikely to be the cause of the malignancy, as both the transgene and surrounding genes were not overexpressed in the blast cells. The identification of somatic mutations associated with AML further suggests that patients with SCD may be at an increased risk of hematologic malignancies, likely due to a combination of risks associated with the disease, autologous transplantation, and disease management processes.^79^

In another research direction, molecular studies have demonstrated that the reactivation of γ hemoglobin, which is replaced by β hemoglobin after birth, can be induced by the repression of the transcription factor B cell lymphoma/leukemia 11A (BCL11A). This process leads to an elevation in fetal hemoglobin levels, which consist of two α and two γ chains, and subsequently restricts the progression of both SCD and TDT, resulting in reduced morbidity and mortality.^80^ Accordingly, a gene editing approach utilizing the CRISPR/Cas9 system to induce DNA double‐strand breaks (DSBs) within the erythroid enhancer of BCL11A has successfully promoted the expression of fetal hemoglobin in both mouse models and primary human progenitors.^81^ This advancement has led to the development of CASGEVY, the first and currently only US FDA‐approved gene editing medicine for rare genetic diseases.^16^ Clinical evidence suggests that shortly after the reinfusion of edited autologous CD34+ cells, allelic editing was observed in the bone marrow and blood of both TDT and SCD patients, accompanied by a significant elevation of fetal hemoglobin in a pancellular distribution.^82^, ^83^, ^84^ More than 90% of treated patients in the TDT group achieved transfusion independence by the endpoint, while 97% of SCD patients were free from vaso‐occlusive crises.^83^, ^84^ Importantly, no evidence of off‐target editing by CRISPR/Cas9 was found in the CD34+ cells in any of these studies, and the common adverse events were also consistent with those observed during the procedure of autologous HSPC transplantation.^82^, ^83^, ^84^

#### 3.2.2 Hemophilia

Hemophilia A and B are both X‐linked disorders caused by mutations in the genes that encode clotting factors VIII and IX, respectively. These mutations result in either a deficiency or dysfunction of the clotting factors, leading to impaired blood coagulation. Frequent intravenous injections of recombinant factors or active analogs can prevent and treat bleeding; however, they impose a substantial burden on patients.^85^ In contrast, gene replacement therapies that deliver long‐lasting exogenous genes encoding the missing clotting factors present a novel curative option, potentially requiring only a single dose of medication for these diseases.

The clinically applied medications HEMGENIX and BEQVEZ deliver genes encoding a high‐activity variant of factor IX (FIX‐R338L), while ROCTAVIAN contains a more effective, shortened variant of factor VIII, specifically the 14‐amino acid SQ variant of B‐domain‐deletion (hFVIII‐SQ), with a gene length suitable for encapsulation in AAV vectors.^86^, ^87^ All these therapies utilize liver‐specific promoters and are administered systemically through various serotypes of AAV vectors.^16^, ^87^, ^88^ Notably, rAAV5 was selected by HEMGENIX and ROCTAVIAN for the delivery of factor IX and factor VIII, respectively, due to its reduced sensitivity to preexisting NAbs.^89^ In contrast, BEQVEZ utilized rAAVrh74 for the delivery of factor IX targeting Hemophilia B, achieving the lowest therapeutic dose of viral vector at 5 × 10^11^ vector genomes (vg)/kg.^90^ The therapeutic efficacy of these three medications was evidenced by a decrease in annualized bleeding rates (ABR) following treatment, alongside significant elevations in in vivo clotting factor activity and reductions in the need for exogenous infusions.^90^, ^91^, ^92^, ^93^, ^94^, ^95^ Improvements in quality of life across several domains were also reported.^96^ However, concerns regarding adverse reactions related to liver function have emerged, primarily due to the potential immunotoxicity associated with the hepatocyte affinity of the viral vector and the liver‐specific expression of exogenous products. Although mild to moderate transaminase elevations were observed in a substantial proportion of participants, long‐term follow‐up—extending to at least 7 years—in these clinical trials, even with dosages reaching up to 6 × 10^13^ vg/kg, demonstrated consistent efficacy with no evidence of sustained or unmanageable hepatic toxicity or tumorigenesis associated with the treatments.^95^, ^97^ Nevertheless, a long‐term evaluation of mature clinical data in factor IX trials is still necessary, as the available long‐term safety data are limited to a small cohort size. Furthermore, the prediction of decreased factor IX due to liver growth in pediatric patients, along with the observed decline in factor VIII activity levels over time in follow‐up studies, underscores the need for more effective gene therapy approaches that exhibit lower immunogenicity and utilize redosable vectors.

### 3.3 Dystrophic epidermolysis bullosa

VYJUVEK (beremagene geperpavec) is currently the first and only approved topical gene replacement therapy for dystrophic epidermolysis bullosa (DEB).^16^ DEB is a rare disorder caused by genetic variations affecting cell adhesion, resulting in extremely fragile skin that is prone to blisters, wounds, and scars.^98^ The major form of DEB is attributed to mutations in the COL7A1 gene, which encodes collagen type VII (COL7), a protein expressed in the basement membrane of the dermis that contributes to the maintenance of skin structural integrity and the development of keratinocytes and fibroblasts.^99^

To deliver and replace COL7 in the cells surrounding the wounds of DEB patients and promote healing, VYJUVEK utilizes a genetically modified, replication‐defective HSV‐1 as the vector to express human COL7 as the therapeutic agent. The medication is combined with a sterile gel to enhance adherence to the wound, thereby stabilizing drug administration.^100^ A double‐blind Phase III trial (NCT04491604) evaluated the efficacy of VYJUVEK in 31 DEB patients, revealing that 46% (95% CI: 24–68, *p* = 0.002) more patients in the treatment group achieved complete wound healing, with only mild erythema considered a treatment‐related adverse event after 6 months. No significant immunologic reactions were detected, even though the study design permitted readministration of the medication up to four times.^101^ An additional Phase I/II trial (NCT03536143) further demonstrated the expression of COL7 in the wounds, providing hope for an improved quality of life for DEB patients.^100^

In parallel with the development of VYJUVEK, similar redosable topical approaches are under investigation, including HSV‐1 delivered TGM1 and SPINK5 for autosomal recessive congenital ichthyosis and Netherton syndrome, respectively. Additionally, a strategy involving the treatment of patient‐derived dermal cells with gene therapy, followed by ex vivo expansion and autotransplantation, is also undergoing clinical research.^102^

### 3.4 Neurological disorders

#### 3.4.1 Spinal muscular atrophy

Spinal muscular atrophy (SMA) is a rare and severe genetic disorder that primarily affects the motor neurons in the spinal cord, resulting in progressive muscle weakness and atrophy, as well as respiratory and swallowing difficulties as the disease progresses.^103^ Approximately 95% of individuals with SMA possess biallelic mutations in the survival motor neuron 1 (SMN1) gene, which encodes the survival motor neuron (SMN) protein essential for the maintenance and survival of motor neurons.^104^ SMA is classified into several types based on the age of onset and the severity of symptoms, which correlate with the copy number of SMN2—a paralog of SMN1 that produces low but functional SMN protein. Consequently, the copy number of SMN2 serves as a predictive indicator of SMA types prior to the onset of symptoms.^105^ Typically, a single copy of SMN2 is associated with fetal‐onset SMA type 0, which has a very limited survival duration after birth. In contrast, the majority of patients with SMA type 1 and type 2 have two and three copies of SMN2, respectively, experiencing rapid neuromuscular decline around the ages of 2 and 13 years.^106^, ^107^

Several therapeutic approaches aimed at enhancing SMN production through either SMN2 or SMN1 are currently available. An antisense oligonucleotide (AON) targeting exon 7 of the SMN2 pre‐mRNA, known as Nusinersen, has been shown to elevate the expression of full‐length SMN protein and has received approval for clinical use.^108^ However, limitations such as the necessity for repeated administration and inadequate delivery to target cells have been persistent challenges associated with this type of treatment. Conversely, the absence of SMN1 presents an ideal target for gene replacement therapy, and the therapeutic efficacy of ZOLGENSMA, an rAAV9 vector containing a fully functional human SMN transgene driven by the CAG promoter, has been demonstrated in several trials. The effects of this single‐dose, intravenously administered systemic gene therapy for patients with SMA type 1 have been showcased in two Phase III trials (NCT03306277, NCT03461289), where a significantly larger proportion of the treated group achieved primary endpoints, with most patients surviving without permanent ventilatory support at 14 months.^109^, ^110^ The efficacy of ZOLGENSMA for SMA types 1 and 2 was also separately analyzed in another Phase III trial (NCT03505099). All enrolled infants predicted to develop SMA type 1 based on the copy number of SMN2 were able to sit independently for more than 30 s within 18 months posttreatment, while all children with SMA type 2 could stand independently before 24 months. None of these patients required permanent ventilation at 14 months.^106^, ^107^ Further subgroup analysis (NCT02122952) indicated that greater benefits could be achieved if gene therapy is administered at an earlier age, while long‐term follow‐up results (NCT03421977) supported the sustained benefits and safety profile up to 6 years of age for SMA type 1 patients.^111^, ^112^

Despite these advantages, immunotoxicity remains a significant concern associated with the systemic administration of AAV capsids, particularly in the treatment of SMA, where a higher dosage of 1.1 × 10^14^ vg/kg is utilized compared with the dosages referenced in the section on hematological diseases (ranging from 5 × 10^11^ to 6 × 10^13^ vg/kg).^113^ A study investigating adverse events among 325 patients who received ZOLGENSMA, including those from the aforementioned clinical trials, revealed that liver‐associated adverse events were reported in 23–34% of patients, with elevated aminotransferases observed in 90% of these cases, and two instances of serious acute liver injury.^114^ It is believed that immunity against the AAV capsid contributes to the rapid onset of hepatotoxicity, which aligns with findings that the highest concentration of vector genomes was detected in the liver of two patients who died from causes unrelated to the gene therapy.^115^ This underscores the necessity for immunosuppressive interventions during the clinical treatment process, as well as the development of new delivery methods characterized by lower immunogenicity, to enhance the safety and efficacy of gene therapy.

#### 3.4.2 Aromatic L‐amino acid decarboxylase deficiency

Aromatic L‐amino acid decarboxylase (AADC) deficiency a CNS genetic disorder caused by biallelic variants in the dopa decarboxylase (DDC) gene located on Chromosome 7, which encodes the AADC enzyme. This enzyme catalyzes the decarboxylation of the intermediates 5‐hydroxytryptophan and l‐dopa to produce serotonin and dopamine in a pyridoxal phosphate‐dependent manner.^116^ The absence of AADC disrupts the synthesis of these monoamine neurotransmitters, leading to hypotonia, movement disorders, developmental delays, and autonomic symptoms.^117^

The exogenous human gene encoding AADC, driven by the CMV promoter, was utilized by KEBILIDI for gene replacement therapy targeting this rare disease. The gene was delivered via AAV2 through bilateral intraputaminal injections. Clinical evidence indicated a rapid improvement in motor function within 12 months, as assessed using the Peabody Developmental Motor Scales, with a median increase of 62 score points (*p* = 0.005).^118^  A long‐term follow‐up study extending over 5 years further demonstrated the enduring therapeutic effects of this approach, evidenced by improvements in patient symptoms, growth, and quality of life, as well as an increase in dopamine production, confirmed through positron emission tomography and neurotransmitter analysis. Subgroup analyses revealed that younger patients experienced greater benefits.^119^ No treatment‐associated brain injuries or immunotoxicities were observed in these trials, and the most common adverse events were manageable dyskinesias, which were classified as mild to moderate.^118^, ^119^ An additional animal study conducted in monkeys compared various administration methods, including intraputaminal, intracerebroventricular (ICV), and intrathecal (IT, lumbar) routes. The results supported the clinical administration strategy, indicating that intraputaminal dosing resulted in the highest levels of exogenous gene expression in the putamen, caudate, and globus pallidus, with undetectable expression in the spinal cord and dorsal root ganglia. Furthermore, intraputaminal administration resulted in no detectable transgene and fewer AAV antibodies in the blood, suggesting a lower likelihood of off‐target effects and immune toxicity.^120^ This provides a reference for gene therapy delivery methods for other rare diseases that present with CNS symptoms.

### 3.5 Duchenne muscular dystrophy

Duchenne muscular dystrophy (DMD) is the most prevalent genetic muscular disorder, arising from genetic frame‐shift mutations in the dystrophin gene, which is the largest gene in the human genome.^121^ This gene contains hotspot regions that are particularly susceptible to deletion; specifically, the distal hotspot region spans exons 44–55, while the proximal hotspot region encompasses exons 2–19, accounting for 85.7 and 14.3% of deletions, respectively.^122^ These mutations lead to the production of an abnormal and truncated protein, which hinders cell regeneration and induces myocyte necrosis.^123^ Similar to SMA, several AONs targeting the mutated exons to induce exon skipping have been approved for clinical application in the treatment of DMD. However, these approaches face consistent challenges, including the need for repeated administration, poor delivery efficiency, ambiguous evidence, and high costs.^22^, ^124^, ^125^, ^126^

ELEVIDYS (SPR9001, delandistrogene moxeparvovec) was a gene replacement method developed for ambulatory patients (4–5 years) with DMD, making it the only US FDA approved gene therapy for genetic muscular disorders at the time of manuscript completion.^127^ This approach employs rAAVrh74 as the vector and the MHCK7 gene regulatory component as the regulatory element. The MHCK7 component is optimized from murine muscle creatine kinase (MCK), comprising a creatine kinase (CK) 7 promoter and an α‐myosin heavy chain enhancer, which contributes to its specificity and high activation efficiency in muscle tissue.^128^ The therapeutic cargo delivered consists of a shortened, functional, codon‐optimized microdystrophin, which includes spectrin repeats 1–3 and 24, as well as hinges 1, 2, and 4.^129^ Clinical evidence indicates the safety and potential for long‐term improvement in muscle function following a single dose of SPR9001 treatment.^130^, ^131^, ^132^, ^133^, ^134^  The expression of microdystrophin, detected by western blot, exhibited robust sarcolemmal localization at week 12 following the single intravenous infusion, with a mean (SD) change from baseline (CFBL) of 54.2% (42.6) (*p* < 0.0001).^132^ Several clinical studies concluded that, compared with a propensity‐score‐weighted external control (EC) cohort, the mean North Star Ambulatory Assessment (NSAA) scores significantly increased for up to 4 years, suggesting a positive effect of this treatment on disease progression.^130^, ^134^

However, controversial opinions suggest that these studies did not achieve a statistically significant difference in NSAA compared with the placebo (95% CI: −0.45, 1.74; *p* = 0.24). Additionally, the correlation between the expression of microdystrophin and functional improvement remains uncertain, highlighting the need for more statistically significant primary endpoints or more robust subgroup analysis results to evaluate therapeutic efficacy.^135^

The most common treatment‐related adverse events observed were mild to moderate vomiting and hepatotoxicity, which occurred within 70 days posttreatment and subsequently resolved.^134^ Drawing from the experiences of these trials, practical considerations have been outlined in a report recommending that anti‐AAV antibody levels, liver function, platelet count, and troponin‐I should be monitored before and after AAV‐based gene therapy. Furthermore, the maintenance of corticosteroid therapy postinfusion is deemed important for managing adverse events.^131^

Moreover, it is evident that numerous therapeutic approaches are currently under investigation in both preclinical and clinical studies.^14^ Among these, the advancements in gene therapy for genetic muscular disorders serve as an exemplary case illustrating the developmental trends in this field. These disorders share significant gene therapy requirements with diseases affecting other systems, owing to the complexity of the molecular pathogenesis underlying muscle diseases, the extensive number of muscle cells that necessitate therapeutic targeting, and the specific targeting requirements of various muscle types.^17^, ^18^, ^19^, ^20^

## 4 THE DEVELOPMENT OF GENE THERAPY APPROACHES IN GENETIC MUSCULAR DISEASES

Mammalian muscle constitutes approximately 40% of total body mass, often necessitating high dosages of vectors in gene therapy.^17^ Genetic muscular disorders encompass a wide range of genetic diseases that lead to muscle weakness, affecting various bodily systems, including movement, breathing, heart function, the endocrine system, and CNS.^137^ The study by Theadoma et al.^138^ examined the prevalence of genetic muscular disorders across four different ethnic groups and identified various types of disorders including muscular dystrophy, congenital myopathy, ion channel muscle disease, Pompe disease, and other myopathies. Muscular dystrophy emerges as the most prevalent genetic muscle disorder, with subtypes such as congenital muscular dystrophy (CMD), DMD, Becker muscular dystrophy (BMD), Emery Dreifuss muscular dystrophy, facioscapulohumeral muscular dystrophy (FSHD), limb‐girdle muscular dystrophy (LGMD), distal muscular dystrophy (DD), oculopharyngeal muscular dystrophy (OPMD), and others categorized based on muscle distribution, clinical features, and age of onset.^139^

### 4.1 Mutational spectrum of genetic muscular diseases

**FIGURE 3:**
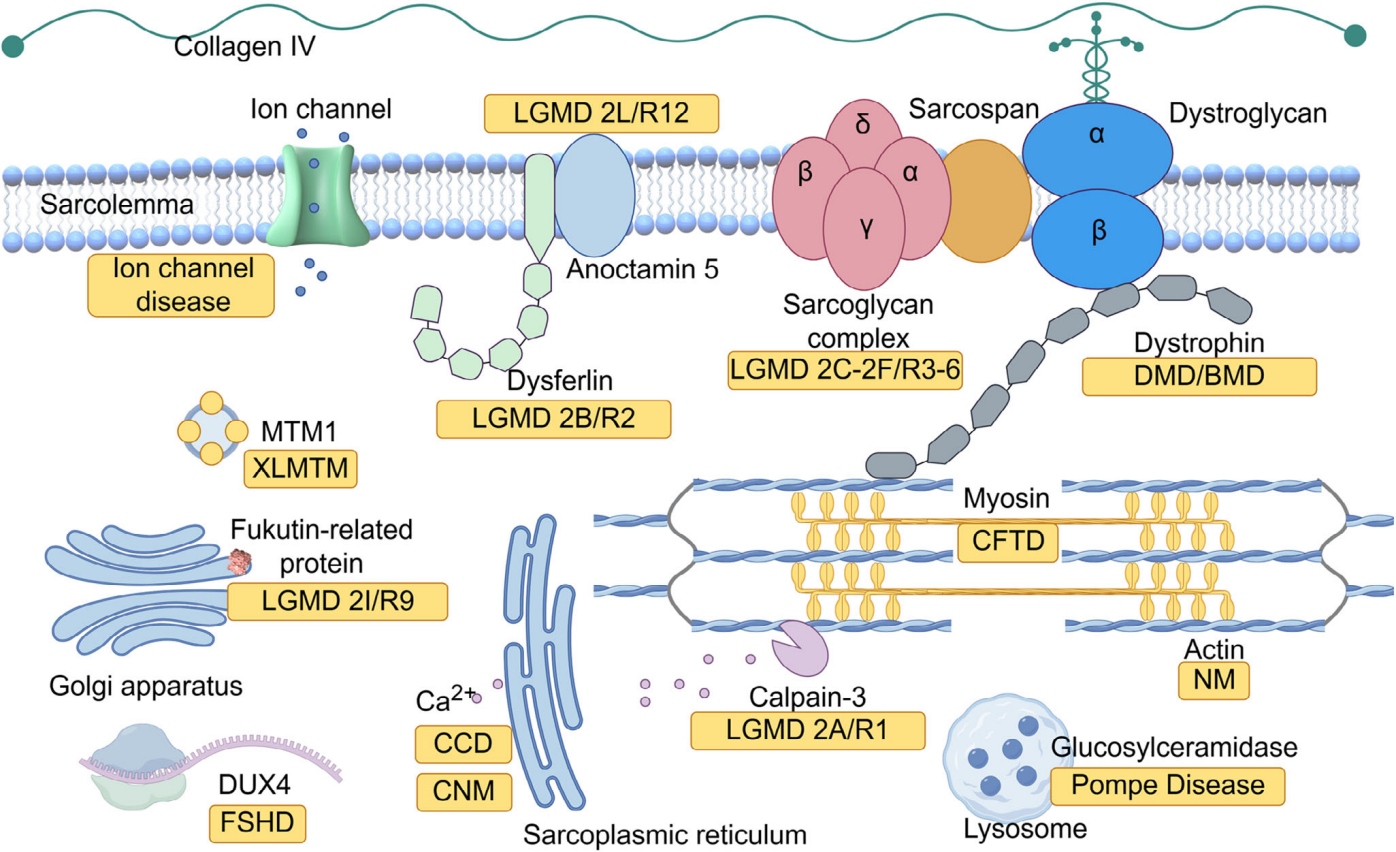
A brief summary of common subtypes of genetic muscular disorders and their respective disease pathogenesis. This includes several representative muscular dystrophies (DMD/BMD, LGMDs, and FSHD), congenital myopathies (CCD, central core disease; CFTD, congenital fiber type disproportion; CNM, centronuclear myopathy; NM, nemaline myopathy; XLMTM, X‐linked myotubular myopathy), ion channel muscle disease, and Pompe disease.

Current molecular biology research has highlighted that numerous genetic muscular disorders are attributed to monogenic diseases, suggesting that gene therapy could serve as an effective treatment strategy (Figure 3).

For instance, dystrophin, as mentioned above, is one of the major components of the dystrophin‐associated protein complex (DAPC).^140^ Monogenic mutations in other subunits of the DAPC can also directly affect dystrophin function, leading to various types of muscular dystrophies, including certain forms of LGMDs.^141^ Alternatively, mutations in other related genes can indirectly contribute to muscular dystrophies; for example, OPMD is caused by a loss‐of‐function mutation in an RNA binding protein,^142^, ^143^ and excessive activation of DUX4 triggers FSHD.^23^

Congenital myopathy, however, encompasses a group of more complex disorders that are less well understood. These diseases can arise from either monogenic or multigenic mutations, but their subtypes are classified according to the morphological features observed in muscle biopsies. Notably, the same mutation may result in different muscle pathologies. Recent knowledge indicates that, irrespective of the distinct morphologies, these mutations commonly disrupt the function of either actin thin filaments, the excitation–contraction coupling apparatus, sarcoplasmic reticulum Ca^2+^ stores, or myosin.^144^

Pompe disease, also known as acid maltase deficiency or glycogen storage disease type II, affects muscle function through a distinctly different mechanism. This metabolic disorder is caused by mutations in the glucosylceramidase (GAA) gene, which encodes the lysosomal enzyme acid alpha‐glucosidase, responsible for catalyzing the conversion of glycogen into glucose. A deficiency of this enzyme results in lysosomal glycogen accumulation, leading to significant pathology in both skeletal and cardiac muscle.^145^

With deepening understanding of these molecular pathogenic mechanisms and advancements in gene therapy methods, numerous genetic muscular disorders are currently undergoing clinical trials involving gene therapy (Table 2), while new treatment options continue to emerge through preclinical research (Table 3). Among these approaches, gene replacement remains the most prevalent strategy in clinical trials. Concurrently, as gene editing technology continues to mature, both in vivo and stem cell‐based ex vivo gene editing techniques are being rapidly implemented in clinical trials for specific types of muscular dystrophies.

**TABLE 2:** Summary of gene replacement and gene editing therapy clinical trials for muscular disorders.

Method	Disease	Therapy	Vector	Promoter/transgene	Delivery method/dosage	Age range	Outcomes	Safety	Refs.
Gene replacement	Duchenne muscular dystrophy (DMD)	SPR9001 (ELEVIDYS; delandistrogene moxeparvovec‐rokl)	rAAVrh74	MHCK7/microdystrophin includes spectrin repeats 1–3 and 24, as well as hinges 1, 2, and 4	Single IV infusion, 1.33 × 10^14^ vg/kg	>2 years	CFBL of sarcolemmal localization at week 12: 54.2% (42.6); *p* < 0.0001 in Western blot; CFBL of NSAA versus natural history control: +3.2 (0.6); *p* < 0.0001;	Vomiting; decreased appetite; glutamate dehydrogenase increased; nausea	NCT04626674^131^, ^132^
					Single IV infusion	<4 years	–	–	NCT06128564
					Single IV infusion	4–7 years	–	–	NCT05096221
					Long‐term follow‐up safety and efficacy study	>0 year	–	–	NCT05689164
					Single IV infusion, 2.0 × 10^14^ vg/kg	3 months to 7 years	Muscle fibers expressing microdystrophin: 81.2%; mean intensity at the sarcolemma: 96%: mean expression by Western blot: 95.8% with adjustment; mean NSAA total score at 4 years: +7.0 (2.9)	Vomiting; elevated liver enzymes; nausea; fatigue; asthenia; decreased appetite	NCT03375164^131^, ^132^, ^133^, ^134^
					Single IV infusion	>0 year	–	–	NCT05881408
					Single IV infusion	4–9 years	–	–	NCT06241950
					Single IV infusion, 2.0 × 10^14^ vg/kg	4–7 years	Mean CFBL in dystrophin localization: significantly increased; mean CFBL in NSAA total score at week 48: significantly increased in selected subgroups	Rhabdomyolysis; Increased transaminases; liver injury; vomiting; nausea; gamma‐glutamyl transferase increased	NCT03769116^130^, ^131^, ^132^
		RGX‐202	rAAV8	Spc5‐12/microdystrophin with functional C‐terminal (CT) domain	A single IV infusion, 1 × 10^14^ and 2 × 10^14^ GC/kg	1–11 years	Increase in dystrophin expression and reduce in CK levels; improvements in daily activities	–	NCT05693142^147^, ^148^
		SGT‐001	rAAV9	CK8/microdystrophin	A single IV infusion, up to 2.0 × 10^14^ vg/kg	4–17 years	Dystrophin levels up to 17.5% by western blot; improvements in 6MWD, NSAA score and pulmonary function	Thrombocytopenia, complement activation, reduced red blood cell count, acute kidney injury, and cardiopulmonary insufficiency	NCT03368742^152^
		SGT‐003	AAV‐SLB101	Muscle‐specific promoter/microdystrophin with R16‐R17 nNOS binding domain	A single IV infusion	4–11 years	–	–	NCT06138639
		PF‐06939926	rAAV9	hMSP/microdystrophin	A single IV infusion	2–3 years	–	–	NCT05429372
					A single IV infusion, up to 2.0 × 10^14^ vg/kg	≥4 years	Microdystrophin expression to 24–50% by immunoaffinity LC–MS; functional improvement;	Dehydration and complement activation, including acute kidney injury, and thrombocytopenia	NCT03362502^152^
					Long‐term follow‐up safety and efficacy study	>0 year	–	–	NCT05689164
					A single IV infusion	4–7 years	–	–	NCT04281485^152^
		JWK007	rAAVrh74	Muscle‐specific promoter/microdystrophin with nNOS and membrane‐binding domains	A single IV infusion, 1 × 10^14^ and 2 × 10^14^ vg/kg	5–10 years	–	–	NCT06114056
	Becker muscular dystrophy (BMD)/sporadic inclusion body myositis (sIBM)	rAAV1.CMV.huFollistatin344	rAAV1	CMV/human Follistatin344	A single leg injection, 2 × 10^13^, 3 × 10^13^, and 6 × 10^13^ vg/kg	≥18 years	Improved 6MWT; reduced endomysial fibrosis, reduced central nucleation, and more normal fiber size distribution	–	NCT01519349^154^
	Limb‐girdle muscular dystrophy, type 2D/R3 (LGMD2D/R3)	SRP‐9004	scAAVrh74	tMCK/human SGCA	Isolated limb Infusion to a single limb or both limbs, 1–3 × 10^12^ vg/kg	≥7 years	–	–	NCT01976091
		rAAV1.tMCK.hαSG	rAAV1	tMCK/human‐SGCA	two to six separate injections intoextensor digitorum brevis, 3.25 × 10^11^ vg/kg	≥5 years	–	–	NCT00494195
	Limb‐girdle muscular dystrophy, type 2E/R4 (LGMD2E/R4)	SRP‐9003	scAAVrh74	MHCK7/human SGCB	A single IV infusion	≥4 years	–	–	NCT06246513
			scAAVrh74	MHCK7/human SGCB	A single IV infusion, 1.85 × 10^13^ and 7.41 × 10^13^ vg/kg	4–15 years	Maintenance of SGCB expression and NSAA score at year 2	Increase in bilirubin, dizziness, decreased appetite, vomiting, nausea, abdominal pain, elevated GGT level	NCT03652259^161^
			scAAVrh74	MHCK7/human SGCB	A single IV infusion	4–50 years	–	–	NCT05876780
	Limb‐girdle muscular dystrophy, type 2I/R9 (LGMD2I/R9)	GNT0006	rAAV9	(Unpublished promoter)/human FKRP	A single IV infusion, 9 × 10^12^ and 2.7 × 10^13^ vg/kg	≥16 years	Decline in levels of creatine kinase; Improved velocity; disappearance of cramps, myalgia and improved quality of life; transgene expression on the 3‐month muscle biopsy	No unexpected safety signal	NCT05224505^164^
		AB‐1003	rAAV9	(Unpublished promoter)/human FKRP	A single IV infusion	18–65 years	–	–	NCT05230459
	Limb‐girdle muscular dystrophy, type 2B/R2 (LGMD2B/R2)	SRP‐6004	rAAVrh74	MHCK7/DYSF splitted in dual vectors	A single IV infusion	18–50 years	–	–	NCT05906251
		rAAVrh74.MHCK7.DYSF.DV	rAAVrh74	MHCK7/DYSF splitted in dual vectors	Bilateral injections with one extensor digitorum brevis muscle, 2 × 10^12^ and 6 × 10^12^ vg/kg	≥18 years	–	–	NCT02710500
	X‐linked myotubular myopathy	AT132	rAAV8	Des/human MTM1	A single IV infusion, 1.3 × 10^14^ and 3.5 × 10^14^ vg/kg	≥5 years	Reduction in least squares mean hours per day of ventilator support from baseline compared with control: 77.7% (95% CI 40.22–115.24, *p* = 0.0002) in lower dose cohort and 22.8% (95% CI 6.15–39.37, *p* = 0.0077) at 24 weeks; 103.7% (95% CI 78.61–128.83, *p* < 0.0001) in lower dose cohort and 62.1% (95% CI 47.49–76.61, *p* < 0.0001) at 48 weeks	Pyrexia, increases in creatine phosphokinase, respiratory tract infections, liver failure, myocarditis	NCT03199469^169^
	Pompe disease	rAAV1–CMV–GAA	rAAV1	CMV/human GAA	A single IV infusion, 1 × 10^12^ and 5 × 10^12^ vg/kg	2–18 years	Maximal inspiratory pressure unchanged; flow and load compensation response with partial/no MV user: increased (*p* < 0.05 to *p* < 0.005) after 180 days	No adverse events related to the study agent, anticapsid and antitransgene antibody response	NCT00976352^173^, ^174^
		AT845	rAAV8	Muscle‐specific promoter/ human GAA	A single IV infusion, 3 × 10^13^, 6 × 10^13^, and 1 × 10^14^ vg/kg	18–80 years	–	Elevated transaminases; no serious adverse events	NCT04174105^183^
		GC301	rAAV9	(Unpublished promoter)/human GAA	A single IV infusion, 3 × 10^13^ and 6 × 10^13^ vg/kg	≥6 years	–	–	NCT06391736
rAAV9	(Unpublished promoter)/human GAA	A single IV infusion, 8 × 10^13^, 1.2 × 10^14^. and 1.8 ×10^14^ vg/kg	≥0.5 years	–	–	NCT05793307
			rAAV9	(Unpublished promoter)/human GAA	A single IV infusion, 1.2 × 10^14^ vg/kg	≥0.5 years	–	–	NCT05567627
		SPK‐3006	Liver‐tropic AAV‐Spark100	hAAT/codon‐optimized secretable GAA	A single IV infusion	≥18 years	–	–	NCT04093349^170^, ^172^
		rAAV9–DES–hGAA	rAAV9	DES/codon‐optimized GAA	A single IV infusion, 4.6 × 10^13^ vg per TA muscle	18–50 years	–	–	NCT02240407
		CRG003	rAAV (Unpublished serotype)	(Unpublished promoter)/human GAA	A single IV infusion	≥18 years	–	–	NCT06178432
		ACTUS‐101	rAAV8	Liver‐specific promoter/human GAA	A single IV infusion, 1.6 × 10^12^ vg/kg	≥18 years	Serum GAA activities from baseline at week 2: 101% to 235%; muscle GAA activity increased at week 52 (*p* < 0.05); not significantly different in 6MWT and upright FVC	Headache, myalgia, and elevated ALT, AST, and GGT	NCT03533673^175^
Gene replacement and gene suppression	Oculopharyngeal muscular dystrophy OPMD)	BB‐301	AAV9PL	Spc5‐12/codon‐optimized PABPN1, and 2 shRNAs directed against the disease‐causing mutant PABPN1	A fixed number of intramuscular injections into the respective pharyngeal constrictor muscles	<65 years	–	–	NCT06185673
Gene suppression	Facioscapulohumeral muscular dystrophy (FSHD)	ARO–DUX4	N/A	–/ARO–DUX4	Single or multiple doses by intravenous (IV) infusion	18–70 years	–	–	NCT06131983
Gene editing	Duchenne muscular dystrophy (DMD)	CRD–TMH‐001	rAAV9	CK8e/dSaCas9–VP64; U6/sgRNA targeting dystrophin promoter	A single IV infusion, 1 × 10^14^ vg/kg	18–28 years	–	Sudden acute respiratory distress 6 days post dose, high levels of IL6, IL8 and MCP‐1, elevated C5b‐9	NCT05514249^177^
		GEN6050X	rAAV9	(Unpublished promoter)/nCas9–CBE; U6/sgRNA targeting exon 50 splicing sites	A single IV infusion, 5 × 10^13^ vg/kg	4‐10 ye‐ars	–	–	NCT06392724
		HG302	rAAV (Unpublished serotype)	(Unpublished promoter) /hfCas12Max–ABE; U6/sgRNA targeting exon 51 splice donor site	A single IV infusion	4–8 years	–	–	NCT06594094
Gene editing and cell therapy	Limb‐girdle muscular dystrophy (LGMD)	GenPHSats–bASKet	Gene edited primary human muscle stem cells	N/A	Injection in the right/left biceps muscle	≥14 years	–	–	NCT05588401

–, Not publicly reported or disclosed.

**TABLE 3:** Representative preclinical research progress of gene therapy for genetic muscular disorders.

Disease	Method	Vector type	Cargo type	Study model	Delivery method	Outcomes	Relevant clinical trial
Duchenne muscular dystrophy (DMD)	Exon skipping	N/A	AONs	mdx mice model	Intracerebroventricular (ICV) delivery	Partial expression of Dp427 in the brain; partial improvements in anxiety traits, unconditioned fear responses, and Pavlovian fear learning and memory^247^, ^248^	–
		N/A	Lipid–ligand‐conjugated complementary strand hybridized with PMOs	mdx mice model	Systemic delivery	Internal deleted dystrophin expression restored; creatine kinase and motor function normalized; cardiac and CNS abnormalities normalized^249^	–
	Gene replacement	Lentiviral vectors	Spc‐5‐12 promoter; coding sequences of four microdystrophin	C57BL/10ScSnDmdmdx/J mice	Intramuscular injection of tibialis anterior muscle (TA) muscles	Dystrophin expression increased, distribution restored, pull force restored, decreased number of mononuclear cells; no detectable integration sites of vectors into the oncogenes^54^	–
		Mymk+Mymg‐lentiviral	CK8e promoter; microdystrophin	mdx mice model	Intramuscular injections and systemic delivery	Specific dystrophin transduction and expression of skeletal muscle, with alleviate pathology in mouse model^250^	–
		AAVMYO2 and AAVMYO3	hDES or SPc5‐12 promoter; microdystrophin	mdx mice model	Systemic delivery	Robust microdystrophin expression and improved muscle function^251^	–
	Gene editing	dual rAAV9	Cbh promoter; Cas9‐N and Cas9‐C; two sgRNAs flanking exon 51	DMDΔ52 pig model; DMDΔ52‐patient iPSC	Intramuscular injection or systemic delivery	DMDΔ51‐52 expression in skeletal muscles, diaphragm and heart; prolonged survival and reduced arrhythmogenic vulnerability^213^	–
		Chemically defined lipid nanoparticle	Cas9 mRNA; two sgRNAs flanking human exon 45	Humanized mouse model with DMDΔ44 and human exon 45	Repeated intramuscular injections	Stable genomic exon skipping and restored dystrophin protein^252^	–
		MyoAAV	Cbh promoter; myospreader SaCas9; two sgRNAs flanking exon 23	mdx mice model	Systemic delivery	1.5 to 2‐fold higher level of exon‐23 deleted mRNA in all muscles in myospreader SaCas9 group than NLS SaCas9 group; 2 to 4‐fold higher level of exon‐23 deleted proteins^253^	–
		rAAV9	CK8e promoter; Cas9; single sgRNA targeting splice acceptor of DMD exon 51	DMDΔ50 mouse or dog models	Intramuscular injection or systemic delivery	Dystrophin protein expression restored throughout skeletal muscles and the heart^217^, ^218^	–
		dual rAAV6	CK8 promoter; Cas9; single sgRNA; HDR template spanning positions X84575274 to X84576081 of the murine DMD gene	mdx mice model	Intramuscular injection of TA muscles or systemic delivery	Dystrophin in up to 70% of the myogenic area; increased force generation; dystrophin expression in skeletal and cardiac muscles after systemic delivery^220^	–
	Base editing	dual rAAV9	Spc‐5‐12 promoter; nCas9‐N and ABE; nCas9‐C; sgRNA targeting splicing sites in human exon 50	DMDΔ5051 mice model with human exon 50	Systemic delivery	>95% dystrophin positive muscle fibers in heart and TA; >75% in diaphragm (DI) muscles; muscle fibrosis and blood creatine kinase activity alleviated; improvement in rotarod running time, forelimb grip strength and cardiac function^180^, ^225^	NCT06392724
		Dual trans‐splicing AAV system	Spc‐5‐12 promoter; nCas9‐N and ABE; nCas9‐C; sgRNA targeting nonsense mutation	mdx mice model	Intramuscular administration into the TA muscle	Converted the premature stop codon to the glutamine codon: 3.3 ± 0.9%; dystrophin restored: in 17 ± 1% of myofibers; no off‐target mutations were detectably^224^	–
	Prime editing	N/A	Spc‐5‐12 promoter; nCas9‐reverse transcriptase; pegRNA targeting nonsense mutation in exon 52	DMDΔ51 iPSC‐derived cardiomyocytes	Nucleofection	+2‐nt GT insertion introduction: 54%; dystrophin protein with respect to the healthy control iPSC‐derived cardiomyocytes: 39.7%^225^	–
LGMD 2A/R1	Gene editing	N/A	Cas9 and mutation‐specific sgRNAs	Primary human muscle stem cells with CAPN3 c.550delA mutant	In vitro delivery	Highly efficient and precise correction of CAPN3 c.550delA to wild‐type by a single cut resulted 5' staggered overhang of one base pair^219^	–
	Gene editing and cell therapy	N/A	CRISPR‐Cas9‐mediated CAPN3 mutations rescued iPSC	C3KO‐NSG mouse combining immunodeficiency and a lack of CAPN3	Transplantation into cardiotoxin‐preinjured TA muscles	New functional chimeric exon without mutations generated in iPSC and iPSC derived CD82+/Pax7+ myogenic progenitor cells; muscle engraftment and rescue of the CAPN3 mRNA in transplanted mice^236^, ^237^	NCT05588401
LGMD 2B/R2	Exon skipping	rAAV2/9	U7 snRNAs	Mouse model with a missense mutant in Dysf	Intramuscular injections into the TA Muscle	Dysf exons 37 and 38 can successfully be skipped in vivo.^254^	NCT04240314
	Gene replacement	Dual AAVrh74	MHCK7 promoter; DYSF5'.PTG and DYSF3'.POLYA	Dysferlin‐deficient mice and nonhuman primates	Intramuscular and systemic delivery	Overexpression of dysferlin restored the function of treated muscle groups measured by membrane repair ability and diaphragm specific force; no safety concerns in nonhuman primates^188^	–
		Dual rAAV9	MHCK7 promoter; DYSF5'.PTG and DYSF3'.POLYA	Dysferlin‐deficient mice	Intramuscular delivery	No statistically significant in proportion of necrotic muscle fibers, muscle fibers with internalized nuclei, and cross‐sectional area of muscle fibers^189^	–
LGMD2D/R3	Gene replacement	scAAVrh74	tMCK promoter; hSGCA	sgca−/− mice	Systemic delivery	Expression of α‐sarcoglycan protein at the sarcolemma membrane: improved the histopathology of limb and diaphragm muscle; reduced serum CK and improved locomotor activity^159^	NCT01976091
LGMD2E/R4	Gene replacement	scAAVrh74	tMCK promoter; hSGCB	Sgcb‐null mice	Isolated limb delivery	Muscle fibers in the lower limb expressing β‐sarcoglycan: 91.2%; improved histological outcomes^160^	NCT03652259
LGMD 2I/R9	Gene replacement	rAAV9	CK8 promoter; full‐length human FKRP cDNA	FKRP P448L mice	Systemic delivery	Restored biochemical defects in a dose‐dependent manner; improvement in the trajectory of disease progression and extension of the expected lifespan^163^	NCT05224505; NCT05230459
		rAAV6, rAAV9 or AAVMYO1	CK8e promoter; human FKRP lacking 5' and 3' UTRs	FKRP P448L mice	Systemic delivery	Improved grip strength in a dose‐ and time‐dependent manner; reduced central nuclei and serum creatine kinase levels; UTR modifications enhanced the translation; high doses of AAV9 or AAVMYO1 exhibited no toxic effects.^184^	–
		rAAV9	CK8e promoter; human FKRP	FKRP P448L mice	Systemic delivery of virus and oral ribitol	More positive matriglycan fibers and pathology improvement in low dose AAV‐FKRP combined with ribitol compared with low‐dose AAV‐FKRP alone^185^	–
OPMD	Gene replacement	rAAV9	Spc‐5‐12 promoter; codon‐optimized PABPN1, and 2 shRNAs directed against PABPN1	C2C12, C. elegans and mouse OPMD model	Intramuscular injection	Robust inhibition of mutant PABPN1 and concomitant replacement of the codon optimized PABPN1 protein.^165^, ^166^	NCT06185673
LAMA2 MD	Gene replacement	N/A	MCK promoter; mag transgene and αLNNd transgene	Mouse model for LAMA2MD	Mating of mice with different genotypes	Fully restored basement membrane stability, recovered muscle force and size, increased overall body weight, and extended life span^193^, ^194^	–
	Gene editing	rAAV9	Cas9 and splice‐site mutation sgRNA targeting exon 2 of LAMA2	Mouse model for LAMA2MD	Systemic delivery	Exon 2 skipping and restoration of full‐length Lama2 protein; improvement in muscle histopathology and function without signs of paralysis^216^	–
GNE myopathy	Gene replacement	AAVrh74	MCK, MHCK7 or CMV promoters; human GNE	Gne−/− hGNED207V Tg mouse, MyoD‐inducible Gne‐deficient cell line, or GNE‐deficient human muscle cell line	Systemic delivery	Long‐term presence and expression of human wt GNE in the murine muscles; some improvements of their mild phenotype; cell models worked well to assay the therapeutic potency in making sialic acid.^195^, ^196^	–
Myotonic dystrophy type 1	Gene suppression	rAAV6	mU6 promoter; HAS siRNAs	Human α‐skeletal muscle actin long‐repeat (HSALR) mouse model of DM1	Systemic delivery	Reduced disease pathology; reduction in the mRNA with expanded CUG repeat, myotonic discharges and myofiber hypertrophy; a shift toward adult pre‐mRNA splicing patterns^206^	–
	Gene editing	N/A	RNP transfection of Cas9 and two sgRNAs flanking CTG‐repeat	DM1–iPSC, DM1–iPSC‐derived myogenic cells, DM1 patient‐specific myoblasts and iPSCs‐derived cardiomyocytes	In vitro delivery	Disappearance of ribonuclear foci in all cell models; MBNL1 restored and splicing pattern of SERCA1 normalized; correction of the underlying spliceopathy^214^, ^215^	–
FSHD	Gene suppression	rAAV6	mU6 promoter; DUX4 miRNAs	Mouse model based on AAV6.CMV.DUX4	Systemic delivery	DUX4 associated myopathy in mouse muscle corrected.^33^, ^207^	NCT06131983
Pompe disease	Gene replacement	Lentiviral vectors	SFFV promoter; IGF2 fused codon‐optimized human GAA	Pompe disease mouse model	Systemic delivery	Completely normalized glycogen levels, pathology, and impaired autophagy in heart and skeletal muscles; normalized glycogen levels and neuroinflammation in CNS; near complete restoration of muscle proteome to wild‐type levels^255^, ^256^	–
		rAAV8	hAAT promoter; sp7‐Δ8‐coGAA	Gaa−/−Cd4−/− mice	Systemic delivery	Viral mediated liver expression of GAA normalized glycogen in all muscle tissues in the mid‐vector and high‐vector dose cohorts and improved muscle strength; glycogen accumulation in brain and spinal cord rescued^172^	NCT04093349
		rAAV8	eMCK promoter; hGAA	Gaa−/−mice; cynomolgus macaques	Systemic delivery	A dose‐dependent increase in GAA activity, glycogen clearance in muscles and heart, and functional improvement in mouse model; anti‐GAA immune response, inflammation, and cardiac abnormalities in nonhuman primates; abnormal processing of human GAA in cynomolgus muscle.^171^	NCT04174105
Issues toward cardiomyocytes	Base editing	rAAV9	Cardiac troponin T promoter; nCas9–ABE; sgRNAs targeting C841 and C844 of CaMKIIδ gene	Cardiomyocytes derived from human iPSCs and a mouse model with ischemia/reperfusion (IR) injury	Injected directly into the area of cardiac injury	Elimination of oxidation sensitive methionine residues confers protection from IR injury and enabled the heart to recover function.^227^, ^228^	–
		Dual rAAV9	Cardiac troponin T promoter; nCas9‐N‐ABE; nCas9‐C; sgRNAs targeting C1208 of MYH7 gene	iPSC derived from patients with hypertrophic cardiomyopathy (HCM), and a humanized mouse model of HCM	Injected through the diaphragm by a subxiphoid approach into the inferior mediastinum	Pathogenic missense variant targeted with minimal bystander editing and little off‐target in iPSCs; the onset of HCM‐mediated pathological remodeling of the heart prevented^229^	–
	Gene editing and cell therapy	N/A	hPSC‐CMs with HCN4, CACNA1H, SLC8A1 knockout and KCNJ2 knockin	Pig model	Transplanted in vivo into uninjured porcine hearts	Transplanted cells engrafted and coupled electromechanically with host cardiomyocytes without causing sustained EAs.^238^	–

### 4.2 Clinical research progress of genetic muscular disorders

#### 4.2.1 Clinical researches on gene replacement

Given that most genetic muscular disorders are characterized by a single pathogenic gene and associated loss‐of‐function mutation profiles,^14^, ^140^, ^144^, ^146^ gene replacement has become a focal point for several subtypes of muscular disorders.

##### Gene replacement trials for DMD

In addition to the approved Elevidys described in the previous section, several other clinical trials targeting DMD utilizing similar strategies have been conducted in recent years. RGX‐202 (NCT05693142) is designed for DMD patients aged 4–11 years and employs the AAV8 vector to deliver the transgene that encodes a novel microdystrophin which incorporates the functional C‐terminal (CT) domain of naturally occurring dystrophin. Preliminary data indicated that RGX‐202 is well tolerated, with no drug‐related serious adverse events reported in three patients. However, further details regarding the adverse events were not published.^147^ More recent data from a poster presentation, which have not undergone peer review, revealed interim results from five patients who received either 1 × 10^14^ genome copies (GC)/kg (Dose Level 1) or 2 × 10^14^ GC/kg (Dose Level 2) of RGX‐202.^148^ The expression levels of microdystrophin in three patients, aged 4, 6, and 10, who received Dose Level 1 were 33.8, 83.4, and 11.1%, respectively, compared with normal controls. Correspondingly, the patients' CK levels were reduced by 43, 94, and 44% from baseline by week 10 postadministration. In the Dose Level 2 group, two patients aged 8 and 12 years exhibited microdystrophin levels of 20.9 and 75.7%, with CK decreases of 90 and 77%, respectively. Additionally, early improvements in daily activities related to strength and function were reported, although these data were not quantified.

However, other gene replacement projects for DMD have encountered challenges. Following two US FDA‐imposed holds on SGT‐001 (NCT03368742) due to serious adverse events, an improved version, SGT‐003 (NCT06138639), which utilizes a proprietary delivery vector (AAV‐SLB101) and a microdystrophin containing the R16‐R17 nNOS binding domain, is scheduled to resume in 2023. Similarly, a randomized, double‐blind, placebo‐controlled Phase III trial of PF‐06939926 (NCT04281485) and a long‐term safety and efficacy assessment (NCT05689164) are currently ongoing, following the unfortunate death of a participant who received a high dose of PF‐06939926 (at least 2 × 10^14^ vg/kg) (NCT03362502).^149^ Currently, additional safety and efficacy data for these latter two approaches are not yet available for peer review; however, the serious adverse events observed in earlier trials warrant concern. A nonhuman primate study of AAV9‐based treatment has indicated that systemic and neuronal toxicity may be general properties associated with the intravenous delivery of AAV vectors at high doses, regardless of the capsid serotype or transgene.^113^ Furthermore, AAV capsids have been reported to directly activate the complement system in a dose‐dependent manner.^150^ Consistently, clinical data from the aforementioned trials, which utilized vector doses of up to 2 × 10^14^ vg/kg, suggested that the adverse events were not linked to altered liver function.^151^, ^152^ Instead, reductions in platelet count, followed by decreases in red blood cell count and transient renal impairment, along with evidence of complement activation, were observed in these trials.^153^ This implies that the innate immune response may be a significant concern during the high‐dose AAV delivery of systemic microdystrophin gene therapy. Therefore, more detailed inclusion criteria—such as preformed antibodies, underlying genetic predispositions of the patients, the characteristics of the AAV capsid, and the degree of purification—should be carefully considered.

In addition to dystrophin, various transgenes have also been investigated in gene replacement clinical trials for muscular dystrophies. Targets related to DAPC, such as follistatin^154^ and GALGT2,^47^ were examined in early trials for DMD and BMD, which is a milder and slowly progressing form of the same disorder.^123^ Notably, a direct bilateral intramuscular injection of alternatively spliced follistatin (FS344), using a CMV promoter delivered via rAAV1 (AAV1.CMV.FS344), demonstrated beneficial effects on the 6‐min walk test (6MWT) index, as well as reductions in endomysial fibrosis and central nucleation, alongside an increase in normal fiber size in BMD patients.^154^ These findings suggest a promising complementary approach for the treatment of DMD.

##### Gene replacement trials for LGMDs

LGMDs comprise a group of muscular dystrophies that are classified into several subtypes, including calpainopathy (LGMD 2A/R1), dysferlinopathy (LGMD 2B/R2), sarcoglycanopathies (LGMD 2C‐2F/R3‐6), dystroglycanopathies (LGMD 2I/R9), and anoctaminopathy (LGMD 2L/R12).^141^, ^155^ As our understanding of the genetic characteristics of LGMD continues to evolve, multiple disease subtypes have emerged as suitable candidates for gene replacement therapy.

LGMD2D/R3 and LGMD2E/R4 are caused by mutations in the α and β‐sarcoglycan genes (SGCA and SGCB, respectively), which lead to tissue damage and vascular spasms.^156^, ^157^ Preclinical evidence has demonstrated that AAV vector delivery, coupled with muscle‐specific promoter‐driven sarcoglycan gene expression, effectively improves the histopathology of limb and diaphragm muscles in mouse models.^158^, ^159^, ^160^ Consequently, several clinical trials have been initiated to investigate the therapeutic efficacy in patients (NCT01976091, NCT03652259, and so on). Interim results from the SRP‐9003 (rAAVrh74.MHCK7.hSGCB) trial (NCT03652259) after a 2‐year follow‐up indicate mild adverse reactions and preliminary motor improvements.^161^ Patients aged 4–15 years with confirmed SGCB mutations at both alleles received a single intravenous infusion of either 1.85 × 10^13^ vg/kg (Cohort 1, *n* = 3) or 7.41 × 10^13^ vg/kg (Cohort 2, *n* = 3) of rAAVrh74, which delivered a codon‐optimized, full‐length human SGCB transgene. Consistent with AAV‐based gene replacement therapies in DMD, the most common treatment‐related adverse events included vomiting and an increase in gamma‐glutamyl transferase levels; however, none of the adverse events were associated with clinical complement activation. Preliminary motor improvements were evidenced by the maintenance of SGCB expression (36.2% in Cohort 1 and 62.1% in Cohort 2) as well as the NSAA score through year 2.^161^

The fukutin‐related protein gene (FKRP) has been shown to play a crucial role in maintaining the glycobiology of α‐dystroglycan, with mutations frequently observed in LGMD 2I/R9 patients.^162^ Animal studies utilizing the FKRP^P448L^ mouse model have demonstrated the therapeutic effects of FKRP gene restoration, resulting in improved disease progression trajectories and extended expected lifespans.^163^ Two related clinical trials, GNT0006 (NCT05224505) and AB‐1003 (NCT05230459), utilized AAV vectors carrying the human FKRP transgene to assess the safety and efficacy of a single intravenous infusion of these therapies. According to an unreviewed report, no unexpected safety signals were identified during the initial phase of GNT0006. Notably, a significant decline in CK levels was observed in three patients, accompanied by improved velocity that was sustained for 1 year, along with evidence of transgene expression noted in the 3‐month muscle biopsy.^164^

##### Gene replacement trials for other muscular disorders

BB‐301 employs a similar yet more complex approach in clinical research for OPMD. OPMD is a rare, autosomal‐dominant, slow‐progressing neuromuscular disorder caused by a polyalanine expansion mutation in the gene encoding polyadenylate RNA binding protein nuclear 1 (PABPN1), a ubiquitously expressed protein that regulates mRNA stability.^142^, ^143^ To introduce DNA replacement encoding a functional protein while compromising the endogenous mutant product, a codon‐optimized wild‐type PABPN1, along with miRNAs targeting endogenous PABPN1, was designed.^165^ Studies utilizing cell and animal models indicated that BB‐301, an AAV9PL‐delivered codon‐optimized gene combined with two siRNAs and driven by a muscle‐specific Spc5‐12 promoter, effectively inhibited the mutant PABPN1. This intervention resulted in a concomitant replacement of the codon‐optimized PABPN1 protein, leading to the restoration of muscle strength and muscle weight.^165^, ^166^ Consequently, a clinical trial assessing the safety of BB‐301 (NCT06185673) has recently been registered. In this trial, patients will receive a single injection of BB‐301 directly into the middle pharyngeal constrictor muscle and the inferior pharyngeal constrictor muscle of the throat, administered through an open surgical procedure under general anesthesia. The results of this trial are still pending announcement.

The strategy outlined serves as a reference for the treatment of various muscular disorders, including congenital myopathy and Pompe disease. X‐linked myotubular myopathy is a life‐threatening congenital muscle disease caused by mutations in the MTM1 gene, resulting in a deficiency of functional myotubularin protein.^167^ AT132 was designed to express a functional human MTM1 gene under the control of the muscle‐specific desmin promoter,^168^ delivered by rAAV8 (rAAV8–Des–hMTM1). Although early‐phase studies demonstrated promising results regarding safety and efficacy in the relatively low virus dose group (1 × 10^14^ vg/kg) (NCT03199469), one patient in the low‐dose group and three patients receiving high‐dose treatment (3 × 10^14^ vg/kg) died during the trial due to severe cholestatic liver failure.^151^, ^169^ This outcome was considered to be related to the innate immune response, as discussed above, suggesting that a re‐evaluation of the safety of high‐dose AAV therapies is warranted.

The monogenic nature of Pompe disease renders it suitable for gene replacement therapy.^170^, ^171^, ^172^ Preliminary clinical data suggest that direct muscle injection of AAV‐mediated GAA gene replacement (NCT00976352, rAAV1–CMV–GAA) may confer benefits to the dynamic motor function of the targeted muscle, as evidenced by greater maximal voluntary ventilation and fewer hours of daily mechanical ventilation compared with the control group.^173^ An additional safety analysis of the trial indicated that there were no adverse events related to the study agent; however, an anticapsid and antitransgene antibody response was observed in all subjects except those who received concomitant immunomodulation, implying a clinically relevant approach to mitigating immune responses to both the AAV capsid protein and the transgene product.^174^ More recently, ACTUS‐101 (AAV8–LSPhGAA, NCT03533673), which contains a liver‐specific regulatory cassette, was reported to increase muscle GAA levels at 52 weeks posttreatment, with no reports of serious adverse reactions or ALT elevations associated with anti‐AAV T‐cell responses at a dosage of 1.6 × 10^12^.^175^ However, considering the potential for anti‐GAA immunity as well as the specific capsid immunity mentioned above, the long‐term safety and clinical benefits of this treatment still require further monitoring across multiple clinical trials utilizing different AAV serotypes and specific promoters.

#### 4.2.2 Clinical researches on gene editing

##### Gene editing trials for DMD

The development of the CRISPR system has provided additional options for gene therapy targeting muscular disorders, and several clinical trials aimed at treating DMD have recently been conducted. The first announced clinical trial for CRISPR therapy in DMD (CRD‐TMH‐001, NCT05514249) employed a CRISPRa strategy, which utilizes an endonuclease‐deficient Cas9 variant (dCas9) fused to the effector domain VP64 to upregulate dystrophin expression following sgRNA targeting of the gene's promoter.^176^ However, limited further details have been reported, aside from the administration of 1 × 10^14^ vg/kg of intravenous rAAV9. Data from a preprint report showed that the treated patient experienced acute respiratory distress and cardiac arrest within 1 week, ultimately resulting in death on day 8 postinjection.^177^ No signs of AAV9 antibodies or effector T cell reactivity were detected, leading researchers to suspect that the high dose of rAAV triggered innate immune signaling, resulting in capillary leak as a manifestation of this toxicity.^177^

Recently, a first‐in‐human, single‐arm, open‐label, single‐center clinical trial (NCT06392724) involving GEN6050X, a base editing tool, was registered. Base editing utilizes nickase Cas9 (nCas9), which cleaves only one strand of DNA, along with a base editing enzyme to induce transitions from A·T to G·C or G·C to A·T.^178^, ^179^ GEN6050X incorporates a cytosine base editor (CBE) that facilitates the G·C to A·T transition and is delivered using dual single‐stranded AAV serotype 9 (ss.AAV9) vectors to modify splicing sites and skip exon 50. The primary endpoints of this study include the safety and tolerability of a single intravenous infusion of GEN6050X in ambulatory boys with DMD. In contrast, HG302 (NCT06594094) employs a similar mechanism but utilizes an adenine base editor (ABE) fused to hfCas12Max, which is smaller than Cas9, and is packaged in a single AAV vector.^180^, ^181^ This approach targets the exon 51 splice donor site, with safety as the primary endpoint. To date, no results have been disclosed for either clinical trial.

##### Gene editing trials for LGMDs

Gene editing can also be applied ex vivo in conjunction with other platforms, such as cell therapy, to provide diverse therapeutic options. Induced pluripotent stem cells (iPSCs), derived from somatic cells through reprogramming technology, facilitate in vivo transplantation and the derivation of patient‐specific cell types, effectively eliminating the immunological issues associated with allotransplantation.^182^ Consequently, gene therapy targeting autologous iPSCs and induced myogenic progenitors ex vivo could lead to the generation of healthy muscle tissue that can self‐renew following transplantation into diseased muscle. This approach circumvents many disadvantages associated with in vivo editing, including delivery limitations, immunogenicity, potential toxicity of foreign proteins, and the dilution of corrected muscle cells due to physiological turnover.^146^ A first‐in‐human application of this strategy was directed against LGDM using gene‐edited primary human satellite cell‐derived autologous muscle stem cells (GenPHSats, NCT05588401). The edited stem cells were injected into the right biceps muscle of the participants. The characterization of the type, incidence, severity, duration, reversibility, and treatability of adverse events will be measured as the primary outcomes, although further details are not yet available.

### 4.3 The development of preclinical research in genetic muscular disorders

The success of a gene therapy product in clinical trials depends on various preliminary data obtained from appropriate pre‐clinical settings. In addition to the preclinical programs associated with the aforementioned clinical solutions, numerous promising gene therapy research initiatives are underway to broaden the range of treatable muscle‐related disorders and enhance treatment techniques (Table 3).

#### 4.3.1 Preclinical studies on gene replacement

As mentioned in the clinical research section, LGMD 2I/R9 is caused by mutations in the FKRP gene, making it a suitable target for gene replacement therapy. One preclinical study investigated the therapeutic potential of an AAV6‐delivered FKRP gene with untranslated region (UTR) modifications. Research conducted in both an LGMD 2I/R9 animal model and a C2C12 cell model suggested that this therapy improved grip strength in a dose‐ and time‐dependent manner, while also reducing central nuclei and serum CK levels by threefold and fivefold, respectively. Furthermore, the UTR modifications enhanced the translation of exogenous genes. Regarding safety, wild‐type mice injected with FKRP packaged in high doses of AAV9 or AAVMYO1 exhibited no toxic effects, further supporting the feasibility of this method.^184^ Additionally, a study explored the efficacy and safety of combining FKRP gene replacement with ribitol, based on findings that ribitol relies on residual FKRP function to restore limited levels of matriglycan. The results indicated that a lower dose of AAV–FKRP (1 × 10^13^ vg/kg) combined with ribitol was safe and more effective than low‐dose AAV‐FKRP alone, thus providing a novel combined approach for gene therapy.^185^

Mutations in the DSFY gene are the primary cause of LGMD 2B/R2, resulting in abnormalities in sarcolemma repair and the formation of new muscle fibers.^186^ To deliver the exogenous DSFY gene via the AAV system, due to the long cDNA,^187^ a dual‐vector system (AAVrh74.MHCK7.DYSF.DV) was developed, in which the DSFY cDNA is divided into two separate vectors (exons 1–31 and exons 22–51) with a 1 kb overlap. This overlap between the two AAV cDNAs facilitates the reconstitution of a split transgene through homologous recombination in the target cells.^188^ Preclinical data have indicated that this strategy resulted in high levels of dysferlin expression without safety concerns in nonhuman primates, as well as functional restoration, reduced inflammatory activity, and mitigation of necrotic processes in dysferlin‐deficient mice. However, another study using AAV9.MHCK7.DYSF.DV did not demonstrate a positive therapeutic effect in mouse models, likely due to the severity of inflammatory changes.^189^  This finding implies that further research with a longer observation period is needed to evaluate in greater detail the effects of such dual AAV vector systems and the delivered transgene on inflammatory components.

LAMA2‐related muscular dystrophy (LAMA2 MD) is a significant subtype of CMD, which encompasses a group of early‐onset muscular dystrophies. It is caused by mutations in the LAMA2 gene, which encodes the α2 subunit of laminin‐211 (composed of α2, β1, and γ1).^190^, ^191^ In mouse models of LAMA2 MD, the expression of laminin‐α4 is compensatorily elevated into adulthood; however, the absence of the laminin N‐terminal (LN) globule results in only weak binding to myotubes.^192^ Studies in animals have demonstrated that in a LAMA2 MD mouse model (dy^W^/dy^W^), transgenic expression of mini‐agrin (mag) and the laminin‐α1 LN‐domain nidogen‐1 (αLNNd) restored laminin's polymerization activity and improved muscle structure, extending the lifespan of the affected mice to as long as 2 years. This finding provides new possibilities for gene replacement therapies to treat LAMA2 MD, should similar mechanisms be confirmed in humans.^193^, ^194^

Additionally, GNE myopathy, an adult‐onset disorder caused by recessive mutations in the UDP‐N‐acetylglucosamine‐2‐epimerase/N‐acetylmannosamine‐kinase (GNE) gene, has shown potential for AAV‐delivered GNE gene replacement in cellular and murine models.^195^, ^196^ The systemic injection of rAAVrh74.MCK.GNE demonstrated the expression of exogenous GNE in Gne−/− hGNED207V Tg mouse muscles, potentially ameliorating their mild phenotype. However, as this mouse model does not exhibit recognized disease characteristics, a more reliable animal model is necessary to confirm the efficacy of this approach.^195^ Furthermore, studies in GNE‐deficient cell models indicated the effectiveness of this strategy in producing Sia, a substrate of the enzyme encoded by GNE, at a relatively low dosage (1 × 10^13^ vg/kg) in vitro.^196^ This suggests the potential of this gene replacement approach for the clinical treatment of GNE myopathy.

#### 4.3.2 Preclinical studies on gene supplementation

Exogenous gene supplementation, without targeting specific gene mutations, has also demonstrated therapeutic potential in various muscular disorders. The administration of E‐Selectin, Estrogen‐related receptor gamma, or IGF‐1 via AAV delivery through intramuscular injection in an animal model of peripheral artery disease has shown improvements in hindlimb perfusion and exercise capacity.^24^, ^25^, ^26^

Additionally, age‐related muscular disorders can benefit from exogenous gene supplementation. Therapeutic administration of an AAV vector encoding human Dok‐7 has been found to suppress muscle denervation, enhance motor function, and improve muscle strength, along with promoting neuromuscular junction innervation in aged mice.^197^ Furthermore, the delivery of AUF1 into muscle fibers has been shown to regulate skeletal muscle mitochondrial oxidative metabolism, restore muscle mass, and may present a therapeutic strategy for sarcopenia.^198^

Expanding the concept of muscle disease treatment reveals that the natural properties of skeletal muscles, which account for 40% of total body weight, make them a promising production hub for the sustained secretion of recombinant proteins, such as coagulation factors and NAbs, into the bloodstream.^17^, ^199^, ^200^ Thus, in addition to directly addressing muscle diseases, gene supplementation therapy targeting skeletal muscle is also appealing for the production of secreted proteins aimed at distal therapeutic targets. Indeed, animal studies have demonstrated benefits in treating obesity and diabetes through AAV‐mediated expression of FGF21 or coexpression of insulin and glucokinase genes in skeletal muscles.^28^, ^29^, ^30^ Furthermore, a case report indicated that the transferred sequence and protein expression persisted for as long as 10 years in a man with severe hemophilia B who received a skeletal muscle injection of a human factor IX‐encoding AAV, highlighting the clinical application potential of this gene therapy strategy.^201^

#### 4.3.3 Preclinical studies on gene suppression

For dominant genetic muscular disorders that involve gain‐of‐function mechanisms, RNAi packaged with AAV vectors presents a promising therapeutic option. Myotonic dystrophy (DM) exemplifies such a disorder. Type 1 and type 2 myotonic dystrophies (DM1 and DM2) are caused by the expansion of CTG repeats in the 3′ UTR of the dystrophia myotonica‐protein kinase gene (DMPK) and CCTG repeats in intron 1 of the CCHC‐type zinc finger nucleic acid binding protein gene, respectively.^202^, ^203^ The expanded repeats do not affect protein translation; however, they lead to the accumulation of nuclear transcripts, which in turn disrupt the localization and activity of RNA‐splicing proteins that regulate the RNA splicing of proteins essential for muscle function.^204^, ^205^ Partial knockdown of both the mutant and nonmutant alleles of DMPK, achieved through intravenously delivered AAV‐packaged siRNA, significantly reduced disease pathology. This included a decrease in CUG repeat expression mRNA, a shift toward adult pre‐mRNA splicing patterns, and reduced myofiber hypertrophy in muscle tissue in a mouse model, suggesting that miRNA therapy may be a viable solution for DM.^206^

DUX4 derepression is widely recognized as the primary contributor to the pathogenesis of FSHD, making it an ideal target for miRNA therapy.^23^ Indeed, in addition to a clinical trial evaluating the treatment effects of an AON (ARO–DUX4) in type 1 FSHD (NCT06131983), preclinical data have provided proof of concept for the AAV‐delivered miDUX4 approach in a mouse model.^207^ A more recent study further assessing the safety issues of this approach indicated that toxicity and off‐target effects of miRNA pose significant challenges for its clinical application. Specifically, one of the miDUX4 sequences resulted in extensive muscle turnover at low doses in the mouse model, while the gene suppression efficiency was significantly lower in human cells.^33^ This suggests that it is crucial for viral‐delivered miRNA therapies to undergo toxicology screening at early preclinical stages.

#### 4.3.4 Preclinical studies on gene editing

##### Gene editing relying on DSBs repairing

One of the fundamental functions of the CRISPR/Cas system is to catalyze DSBs and induce nonhomologous end joining (NHEJ). This process employs proteins that recognize, resect, polymerize, and ligate the DNA ends in a manner that lacks high fidelity, resulting in random deletions and insertions (indels) around the break site.^208^ By utilizing NHEJ induced by DSBs located between two sgRNAs within out‐of‐frame exon(s), this gene editing strategy can be effectively integrated into the exon skipping approach for DMD at the genomic level. Proof‐of‐concept studies published as early as 2016 provided evidence for the use of viral delivery of CRISPR‐based genome editing as a potential therapeutic strategy for restoring skeletal and cardiac muscle function in human myoblasts and mouse models.^209^, ^210^, ^211^, ^212^ These findings were further corroborated by analyses conducted on large animal and human models. In a pig model lacking exon 52 of DMD, both intramuscular and systemic applications of Cas9 and a pair of sgRNAs flanking exon 51, packaged in AAV9 (AAV9–Cas9–gE51), demonstrated the expression of a shortened dystrophin and improved muscle function. A similar result was observed in the corresponding patient‐derived iPSC model with AAV6–Cas9–gE51 application.^213^ This dual sgRNA‐based strategy may also offer benefits for other muscular disorders. Notably, CTG‐repeat expansion in the 3' UTR from DM1 iPSCs has been reported to be successfully excised using this system. RNA sequencing and alternative splicing analyses of the edited iPSCs further demonstrated the reversion of the underlying spliceopathy in DM1 cardiomyocytes.^214^, ^215^ Similarly, in a mouse model of CMD type 1A caused by a splice‐site mutation that led to the exclusion of exon 2 from Lama2 mRNA, a pair of AAV‐delivered sgRNAs restored the functional splice donor site and the expression of full‐length laminin‐2.^216^

Other strategies based on the CRISPR/Cas system‐induced NHEJ are also under investigation. For example, a single‐cut strategy utilizing only one sgRNA and an indel has been shown to be effective, either by disrupting a splicing motif to skip an exon or by reframing a transcript. In a mouse model exhibiting deletion of DMD exon 50, systemic delivery of AAV‐packaged Cas9 along with a single sgRNA resulted in reframing mutations and facilitated the skipping of exon 51.^217^ Similarly, a study in a canine model with exon 50 deletion suggested that systemic injection of AAV9 delivering Cas9 and sgRNA targeting exon 51 restored dystrophin expression in both skeletal and cardiac muscles.^218^ This strategy could also be applicable to other genetic muscular disorders. The targeted design of sgRNA directed towards CAPN3 c.550delA, combined with the delivery of Cas9 in primary human muscle cells from LGMD patients, demonstrated mutation‐specific targeting that yielded highly efficient and precise correction of the open reading frame (ORF), leading to the expression of CAPN3.^219^

The development of CRISPR/Cas systems has also provided multiple precise options for gene editing. The homologous‐directed repair (HDR) strategy, in which DSBs are repaired with the guidance of a dystrophin homology region, was tested in a DMD mouse model to fully correct the mutation. Although there was debate regarding which type of muscle cells were responsible for the HDR events, the presence of HDR‐derived transcripts was detected in the study, highlighting the need for optimization of this strategy.^220^

However, all the gene editing tools mentioned above rely on the generation of DSBs in the genome. The potential for off‐target effects, as well as the occurrence of random indels at the cutting site, may lead to small mutations, large deletions of target loci, or even extensive chromosomal rearrangements.^221^  The long‐term in vivo safety of these approaches for muscular disorders requires careful observation. Furthermore, technological advancements aimed at increasing overall editing efficiency and enhancing the proportion of intended gene modifications—through optimization of delivery methods and gene editing strategies—should be prioritized. Additionally, the insertion of AAV genomes into the host genome via CRISPR‐induced DSBs has been detected more frequently than previously anticipated, highlighting the need to consider potential genotoxicity risks in long‐term and larger‐scale studies.^222^

##### Base editing

The recently developed base editing and prime editing approaches, which do not rely on DNA damage response mechanisms,^178^, ^179^, ^223^ are also being utilized in DMD gene therapy. One study employed CRISPR‐guided ABEs to target a premature stop codon in exon 20 of a mouse model, resulting in a precise A‐to‐G base conversion with a frequency of 3.3 ± 0.9% 8 weeks postadministration, and restored dystrophin expression in 17 ± 1% of myofibers.^224^ A similar approach was utilized in another study, which targeted the splice acceptor or splice donor site of exon 50, successfully promoting exon skipping and restoring dystrophin expression in human iPSC‐derived cardiomyocytes containing exon 51 mutations.^225^  The significant potential of base editing is also highlighted in various other genetic muscular disorders. Modification of oxidation‐sensitive Met281 and Met282 to amino acids that are less susceptible to oxidation has been reported to prevent the overactivation of CaMKIIδ and to protect cardiomyocytes from ischemia/reperfusion (IR) injury.^226^ In this context, CRISPR–Cas9 ABE targeting the codons of these two residues has been shown to convert ATG to GTG codons with efficiencies of 75, 17, and 8% for homozygous, heterozygous, and wild‐type colonies, respectively. Furthermore, cardiac function recovery after IR has been observed in both mouse and humanized mouse models.^227^, ^228^ Additionally, the dominant‐negative c.1208G>A (p.R403Q) variant in β‐myosin (MYH7), a well‐characterized pathogenic variant associated with increased cardiac contractility and the onset of hypertrophic cardiomyopathy, was efficiently corrected using CRISPR‐Cas9 mediated ABE, demonstrating minimal bystander and off‐target effects.^229^ This highlights the potential of base editing as a therapeutic strategy for muscular disorders caused by monogenic variants.

However, while guided by sgRNAs, it is important to acknowledge that base editing still has the potential to induce off‐target events, which are more frequently observed in CBEs.^230^ Furthermore, the base editor, when fused to the Cas protein, is limited to modifying a short range of bases upstream of the PAM at the targeting site, which restricts its application in gene therapy.^231^ Notably, to mitigate or at least lessen this limitation, a machine learning model trained on the sequence‐activity relationships of base editors and their targets in mammalian cells has been reported to identify previously unpredictable C‐to‐G and C‐to‐A editing single nucleotide variants, suggesting a novel strategy for targeting previously intractable sequences.^232^

##### Prime editing

Alternatively, the prime editing system offers a method to introduce a desired sequence of edits without inducing DSBs. This system comprises a nCas9 enzyme fused with a reverse transcriptase, along with a specialized prime editing guide RNA (pegRNA). The pegRNA includes a traditional sgRNA, a reverse transcription template containing the desired edit, and a primer binding site that attaches to the nontarget strand. The reverse transcription template can be programmed to facilitate base replacement, as well as insertions and deletions of nucleotides of varying lengths.^223^ The prime editing approach for DMD has also been tested in exon 51‐deleted iPSC‐derived cardiomyocytes. An sgRNA targeting the premature stop codon in exon 52 guided the insertion of +2‐nt AC nucleotides with an efficiency of 20.2%, and the correct reframing of the ORF was confirmed by the restoration of dystrophin protein in a single clone of iPSC‐differentiated cardiomyocytes.^225^ Despite these advancements, a common challenge for both base editing and prime editing systems is their large size, which exceeds the packaging limitations of AAV. This issue urgently requires attention through improvements in AAV packaging strategies and the development of novel vectors. Additionally, it is important to note that the efficiency of insertion mediated by prime editing is inversely proportional to the length of the insert sequence.^233^

##### Ex vivo gene editing

Along with the advancements in in vivo gene editing, significant progress has also been made in ex vivo approaches for genetic muscular disorders. For example, mutations in the CAPN3 gene have been linked to LGMD2A/R1, while AAV‐mediated CAPN3 gene replacement therapy has been reported to cause severe toxicity due to inadvertent leakage into the heart.^234^, ^235^ To enhance the delivery and maintenance of CAPN3 gene expression specifically within skeletal muscle, a stem cell‐based therapy utilized CRISPR/Cas9‐edited iPSCs derived from three LGMD2A/R1 patients with mutations downstream of exon 15. These iPSCs were modified at exon 14, followed by HDR of the DSB using a template encoding exons 15–24 to bypass the mutations. In vitro analyses, along with data from an immunodeficient mouse model lacking CAPN3 that was transplanted with gene‐corrected myogenic progenitors, demonstrated successful muscle engraftment and restoration of CAPN3 mRNA levels.^236^ A similar study targeting two mutations in exons 3 and 4 of CAPN3 (W130C, 550delA) further underscored the clinical potential of the iPSC gene editing strategy for LGMD2A/R1 patients, employing CRISPR/Cas9‐mediated single DSBs and a wild‐type CAPN3 template to guide HDR.^237^ Human PSC‐derived cardiomyocytes (hPSC‐CMs) represent a promising cell‐based therapy for myocardial infarction, and the editing of ion channel genes using the CRISPR/Cas9 system has demonstrated benefits in this context. The disruption of depolarization‐associated genes HCN4, CACNA1H, and SLC8A1, combined with the overexpression of the hyperpolarization‐associated gene KCNJ2, effectively eliminated the occurrence of transitory ventricular arrhythmias following cell transplantation and enhanced electromechanical coupling with host cardiomyocytes.^238^ Ex vivo delivery offers greater safety compared with in vivo approaches, as patients are not exposed to the gene editing tool. However, the in vitro cultivation and amplification of the targeted cells from the patient after genetic manipulation, while ensuring sufficient cell re‐engraftment, pose challenges that limit this approach to specific conditions.

##### Mitochondrial gene editing

Apart from genomic mutations, the accumulation of mitochondrial dysfunction contributes to energy deficits and alters the balance between protein synthesis and degradation in skeletal muscle, which is associated with skeletal muscle atrophy.^239^, ^240^ Among the clinically confirmed pathogenic mitochondrial DNA (mtDNA) mutations, point mutations account for the vast majority (90 out of 95, or 94.7%), representing potential therapeutic targets for base editing.^241^ However, CRISPR/Cas‐mediated base editing has faced challenges in this application area due to the limited delivery of sgRNA to mitochondria and the lack of efficient mtDNA repair mechanisms.^242^ DddA, a newly described interbacterial toxin, catalyzes the deamination of cytidines within double‐stranded DNA. The fusion of engineered split‐DddA halves, inactive unless reunited and nontoxic, with transcription activator‐like effector (TALE) array proteins and an uracil glycosylase inhibitor (UGI) result in RNA‐free DddA‐derived CBEs (DdCBEs). These editors effectively catalyze C‐to‐T conversions in human mtDNA, exhibiting high target specificity and product purity.^243^ Alternatively, custom‐designed TALEs, composed of TALE DNA‐binding arrays, a catalytically impaired full‐length DddA variant or split DddA halves, and an engineered deoxyadenosine deaminase derived from the E. coli TadA protein, have demonstrated high efficiency in human cells, catalyzing A‐to‐G conversions.^241^ Subsequently, a mitochondria‐localized TALE binding with nickases, along with either the single‐stranded DNA‐specific adenine deaminase TadA8e or the cytosine deaminase APOBEC1 and UGI, has been reported to achieve A‐to‐G or C‐to‐T base editing with up to 77% efficiency and high specificity.^244^ These laboratory research results provide tools for the precise manipulation of mtDNA, with broad implications for the study and potential gene editing therapy of mitochondrial disorders. Nonetheless, several challenges remain before clinical application. mtDNA is a multicopy genome, and the mutational load significantly influences the pathogenicity of heteroplasmic mtDNA mutations.^245^ Therefore, comprehensive evidence regarding in vivo delivery and editing efficiency should be further explored. Conversely, while the off‐target editing of mtDNA may be less critical due to the numerous mitochondria present within a single cell, the potential for substantial nuclear off‐target mutations must still be taken into account.^246^

## 5 OPEN ISSUES AND FUTURE PROSPECTS

**FIGURE 4:**
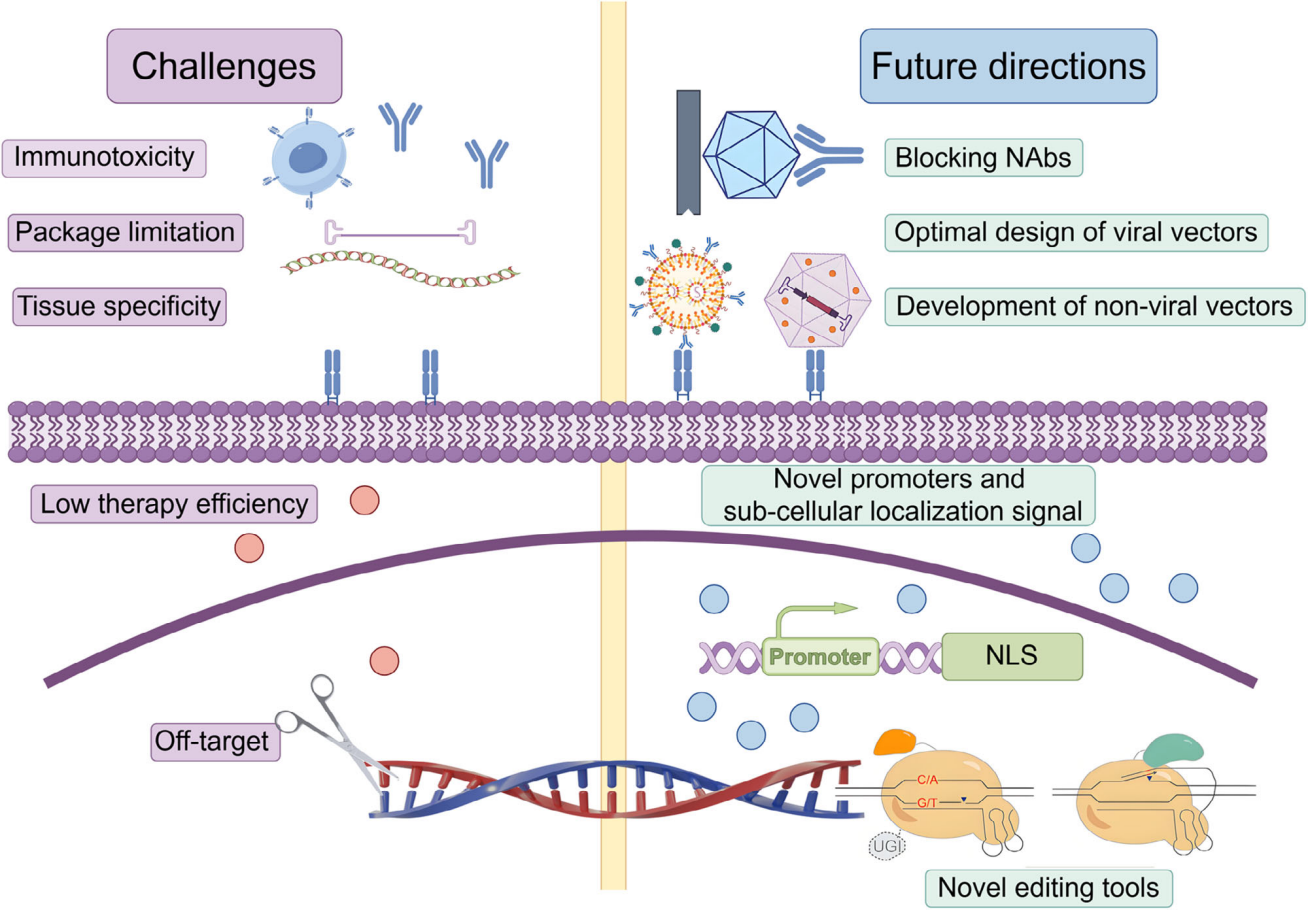
Infographic of challenges and future directions in gene therapy for genetic diseases. Challenges in the therapies include limitations in packaging, immunotoxicity associated with viral capsids and therapeutic cargo, insufficient tissue and subcellular specificity, and off‐target effects observed in gene editing techniques. Emphasized areas for future research include the development of novel packaging strategies and vectors, suppression of immune activity, and enhancements in delivery, expression, and targeting specificity through innovative design technologies.

Despite the promising advancements in preclinical studies, only limited clinical data are currently available, and significant challenges remain in translating these approaches into clinical practice for most muscular disorders. One of the major issues is the limitation of the delivery system. rAAV vectors have emerged as the preferred choice in clinical trials due to their minimal pathogenicity, ability to establish long‐term gene expression, and affinity for muscle tissue.^44^  However, the upper limit of packaging restricts their application in gene therapy, particularly in gene editing. More importantly, safety concerns such as innate and adaptive immune responses associated with high‐dose viral therapies in humans—which are necessary for muscle therapy due to the high proportion of muscle tissue in the body—continue to be a significant issue. The immunotoxicity of the therapeutic cargos, particularly extrinsic gene editing tools like Cas9, is also regarded as a potential safety concern. Additionally, off‐target effects in gene editing methods represent a key obstacle to their clinical application. Various measures have been taken across different research fields to address the challenges currently faced by gene therapy for muscular disorders. These include the development of novel packaging strategies and vectors, suppression of immune activity to reduce vector toxicity, and enhancements in delivery, expression, and targeting specificity through innovative design technologies. (Figure 4)

### 5.1 Novel strategy of AAV package

To address the limitations of packaging capacity during AAV‐mediated therapy, mini‐proteins have been developed for the treatment of DMD. However, this strategy may not be universally applicable to other genetic disorders characterized by large genes. In many gene therapy approaches that utilize long fragment nucleic acids as delivery vehicles—such as base editing, prime editing, or gene replacement that requires full‐length cDNA—the packaging capacity of AAV vectors is often exceeded.^188^, ^224^, ^225^

To overcome this limitation, a dual AAV delivery system has been established. For instance, a study divided CBE or ABE components into N‐terminal and CT halves. Each half was fused with a fast‐splicing split‐intein and packaged into separate AAVs. When coexpressed through the coinfection of AAV particles, the components spliced in trans to reconstitute the full‐length base editor.^257^, ^258^ The therapeutic editing efficiency was demonstrated in the mouse brain, liver, retina, heart, and skeletal muscle, presenting an innovative approach for AAV‐mediated in vivo gene therapy utilizing relatively large editing tools.^258^ However, it is important to note that the nature of dual vector systems necessitates higher titers of viral vectors, which may be less effective than single virus systems and could pose increased immunological and toxicological concerns.

Spliceosome‐mediated RNA trans‐splicing (SMaRT) offers an alternative therapeutic strategy for monogenic diseases characterized by mutations near the 5' or 3' exons.^259^, ^260^ This approach targets RNA at the pre‐mRNA level by utilizing pre‐trans‐spliced molecules (PTMs) that incorporate the wild‐type coding sequence, a robust splicing site, and a binding domain to ensure the specific binding of PTMs to the splicing site of the targeted pre‐mRNA. The PTMs facilitate the incorporation of the WT coding sequence into the corresponding mRNA by obstructing the RNA splicing of the mutant exon on the pre‐mRNA. An in vitro study utilizing primary myotubes derived from a mouse model of LMNA‐related CMD demonstrated that a PTM targeting intron 5 of Lmna pre‐mRNA effectively rescued the mutant phenotype. However, further development is necessary to improve the efficiency of such AAV‐delivered in vivo therapies.^261^

### 5.2 Immunotoxicity of the AAV capsids

Achieving durable and efficient gene therapy in patients with muscular disorders necessitates high doses of AAV vectors as the high proportion of muscle mass. However, this requirement for elevated vector dosages presents significant challenges regarding the safety of AAV‐based gene therapies, as evidenced by serious adverse events reported in several clinical trials.^149^, ^151^ Such adverse events, which range from transient vomiting and nausea to more severe occurrences such as hepatotoxicities, thrombocytopenia, renal impairment, and anemia, were frequently observed in the early stages of multiple AAV trials. These events are highly likely to be associated with innate immune reactions related to systemic doses of AAV capsids.^113^, ^262^ Nevertheless, further research is needed to investigate the detailed mechanisms involved, given the varying immunosuppression strategies employed in different trials, gene therapy modalities, and diseases. Additionally, wide variations in the quantification of vectors may still exist in current clinical trials.^263^ It is essential to develop standardized protocols for collecting and analyzing higher quality data sets across different sponsors to facilitate the development of safe and effective clinical trials.

On the other hand, many humans possess pre‐existing NAbs to AAVs that are induced by natural infections during childhood. The seroprevalence rates of these antibodies vary significantly according to different serotypes, geographic locations, and across various reports.^264^ In a study that defined seropositivity as a neutralizing titer of greater than or equal to 3.1, antibodies against AAV serotypes 2, 5, 6, and 8 were detected in 100, 36, 91, and 90% of subjects, respectively.^265^ Another study, which analyzed a broader range of serotypes, revealed that the prevalences of total immunoglobulin G (IgG) against anti‐AAV1 and ‐AAV2 were higher (67 and 72%) compared with those against anti‐AAV5 (40%), anti‐AAV6 (46%), anti‐AAV8 (38%), and anti‐AAV9 (47%).^266^ These pre‐existing NAbs can potentially inhibit the efficacy of AAV‐based gene therapy. Furthermore, the administration of rAAV vectors in immunologically naive patients can lead to the neutralization of a subsequent injection of the AAV vector due to the generation of NAbs from acquired immunity.^55^, ^56^ Additionally, some NAbs have been reported to cross‐react among different AAV serotypes, further limiting options for sequential gene transfer.^267^ Several strategies are currently under investigation to address these challenges, including immune suppression to block antibody formation, depletion of NAbs, and the engineering of novel vectors.

#### 5.2.1 Antibody formation blocking

Utilizing chemicals to target B and T cells at the appropriate time during AAV mediated gene therapy has emerged as one of the most common immune suppression strategies in recent years. For instance, a study investigating this therapy for hemophilia in nonhuman primates revealed that, in addition to administering mycophenolate mofetil and rapamycin, only a delayed delivery of antithymocyte globulin 5 weeks post‐AAV administration effectively suppressed the development of human factor IX antibodies. This finding underscores the critical importance of timing in T cell‐directed immune suppression for determining transgene product immunogenicity or tolerance.^268^ Furthermore, several agents, including prednisolone, rituximab, sirolimus, rapamycin, and anti‐CD20 antibodies, have been employed either alone or in combination to deplete B cells and the corresponding NAbs.^269^, ^270^, ^271^, ^272^ However, it is important to note that such immune suppression affects the entire body, necessitating careful consideration of associated risks, such as infection.

#### 5.2.2 Antibody depletion

Direct depletion of NAbs also presents a viable approach. IdeS and IdeZ, two homologous cysteine endopeptidases derived from the IgG‐degrading enzyme, have been identified in different species of Streptococcus and possess the ability to cleave human IgG with strict specificity, thereby eliminating Fc‐dependent effector functions.^273^, ^274^ The efficacy of these enzymes in depleting anti‐AAV NAbs has been demonstrated in nonhuman primate models in vivo, as well as in human plasma samples in vitro, offering an additional potential solution to address pre‐existing antibodies.^275^, ^276^ Another method for depleting NAbs is plasmapheresis, which involves the separation of plasma from blood cells using an extracorporeal device. Earlier studies involved discarding the plasma and replacing it with alternative fluids. A human study indicated that frequent sessions of plasmapheresis resulted in a significant reduction of NAbs against several AAVs,^277^ while an animal study further demonstrated successful AAV gene transfer of microdystrophin postplasmapheresis.^278^ Progressive methods that combine plasmapheresis with immunoadsorption columns have since been developed to address the limitations of the original strategy, such as the need for multiple rounds of plasma replacement and the loss of whole antibodies. These immunoadsorption columns, which are loaded with empty AAV capsids, can selectively deplete anti‐AAV NAbs while allowing the transfusion of the remaining antibodies back with the plasma.^279^ This approach supports the administration of AAV‐mediated gene therapy in patients with pre‐existing NAbs, as well as the repeated administration of such therapy.

#### 5.2.3 Engineering of novel vectors

The growing understanding of the AAV‐induced immune response presents opportunities to rationally engineer the specific regions of AAV that are recognized by the immune system, aiming to evade existing NAbs. For instance, the rational design of AAV2, which incorporates a single mutation at residue 265 of VP1, has demonstrated the ability to escape NAb activity while maintaining cellular transduction capabilities.^280^ Additionally, the use of chimeric capsids that combine residues from different serotypes offers another strategy for rational engineering. AAV2.5 was created from the AAV2 capsid by incorporating Q263A, T265 (insertion), N706A, V709A, and T717N from AAV1, resulting in a 2–20‐fold reduction in NAb titer compared with AAV2, while preserving cell tropism.^281^, ^282^ However, it is evident that this strategy is dependent on existing knowledge and is relatively low throughput.

Moreover, novel AAV vectors can be developed through directed evolution with high throughput methodologies. Techniques such as error‐prone PCR,^283^ random peptide display,^284^ and DNA shuffling^285^ have been employed to generate AAV capsid libraries. Their efficacy as gene therapy vectors is assessed positively based on cell delivery efficiency and negatively based on NAb‐positive serum. In one study utilizing this random mutagenesis approach, two variants of AAV5 were selected, exhibiting ten times greater efficiency than the wild‐type in transducing primary human hepatocytes, while demonstrating low seroreactivity.^286^

With the advancement of computer algorithms, machine learning, which excels at managing the relationships among complex factors, is increasingly applied in the medical field. This includes areas such as medical image analysis, disease prediction, and drug discovery. Data related to virus‐mediated immune responses, such as sequences of AAV capsids, B cell‐encoded virus‐specific antibodies,^287^ and epitopes of these antibodies,^288^, ^289^ are continuously accumulating. Consequently, predicting the relationships between AAV sequence features, three‐dimensional structures, and functional properties—such as in vivo immune responses, which are essential for capsid design—falls within the scope of machine learning applications. For instance, machine learning applied to data from AAV capsid libraries collected before and after viral assembly successfully predicted whether unknown sequences could assemble into functional capsids.^290^ Additionally, a study aimed at selecting capsids with high transduction efficiency through a generalizable machine learning model accurately predicted the biodistribution of AAV capsid variants in macaques. The top candidates from their libraries demonstrated up to 1000‐fold greater transduction efficiency in human hepatocytes compared with AAV9.^291^ Therefore, it is reasonable to conclude that, with the appropriate dataset, biological feature selection, model selection, and training approaches, machine learning has the potential to significantly enhance both the yield and throughput of novel AAV vector design, achieving high tissue specificity and low immunogenicity.

### 5.3 Nonviral vectors

Alternatively, the development of novel nonviral vectors, which possess a high capacity for carrying genetic material, offers improved safety, convenient accessibility, and the potential for large‐scale production. These delivery approaches may provide an effective means to address the immunotoxicity and packaging limitations associated with viral vectors. Recent discussions have highlighted various aspects of nonviral vectors applied in gene therapy for genetic muscular disorders.^58^ Generally, polymer nanoparticles, LNPs, inorganic nanocarriers, and nucleic acid conjugates are promising candidates for delivering gene therapy cargos to muscle tissues.^292^, ^293^, ^294^, ^295^, ^296^, ^297^ Some recent advancements are outlined below as examples.

The cell‐internalization SELEX (Systematic Evolution of Ligands by Exponential Enrichment) approach can be employed to select RNA aptamers that bind to and internalize within cells, thereby providing a valuable tool for recognizing and delivering linked cargos into muscle cells.^298^ After 15 cycles of skeletal muscle cell‐internalization SELEX, a screened RNA aptamer labeled A01B was found to preferentially internalize in skeletal muscle cells both in vitro and in vivo while exhibiting decreased affinity for off‐target cells.^299^  This finding suggests a novel strategy in which the A01B RNA aptamer, when fused to short therapeutic AONs,^300^ enhances the targeting capability of AONs against muscular dystrophies. Similarly, the improved targeting ability of AONs fused with cell‐penetrating peptides and antibodies, or linked with stereochemical backbones, is also under investigation.^301^, ^302^, ^303^ These naked oligonucleotides encounter significant challenges in their delivery to the heart, diaphragm, and CNS. A recent study indicated that the use of lipid‐ligand‐conjugated complementary strands, when hybridized with these cargos, markedly enhanced delivery efficiency in a DMD mouse model, effectively normalizing cardiac and CNS abnormalities without any adverse effects.^249^

Furthermore, nonviral vectors have demonstrated the capability to deliver relatively long DNA cargos in gene therapy targeting muscular disorders. A pH‐dependent ionizable lipid with three hydrophobic tails has been reported to formulate a LNPs delivery system that successfully delivered a CRISPR/Cas9‐mediated editing tool into the skeletal muscle of a humanized DMD mouse model, effectively rescuing the loss of dystrophin protein. Importantly, the LNP system can be repeatedly administered intramuscularly, leading to cumulative recovery of dystrophin protein.^252^ Extracellular vesicles (EVs), defined as heterogeneous populations of membrane‐bound vesicles released by various cell types, are also considered candidate gene therapy vectors due to their low toxicity, low immunogenicity, and high loading capacity.^304^, ^305^ A recent study demonstrated the specific and rapid disruption of miR‐29b expression using an artificially engineered EV‐based delivery system, which employed CRISPR/Cas9 to target miR‐29b (EVs–Cas9–29b), and observed a protective effect in an induced muscle atrophy mouse model.^306^ The delivery capacity of other nanoparticles has also been investigated. A calcium phosphate (CaP) biomineralization system has been reported to efficiently load ribonucleoprotein complexes of Cas9 protein and sgRNA (RNPs) to form nanoparticles with good serum stability.^307^ Cellular uptake, nuclear transport, and subsequent DMD gene editing were observed in this study, suggesting a stable and safe nonviral strategy for gene therapy in muscular disorders.^308^ Moreover, nonviral vectors can be combined with AAV capsids to mitigate the clearance of AAV caused by NAbs in vivo. EVs containing packaged AAV have demonstrated the ability to reach the CNS and exhibit greater resistance to NAbs compared with unprotected AAV.^309^ In another study, AAV capsids were modified with unnatural amino acids to which oligonucleotides were ligated. These oligo‐AAVs could be identified by specific sequences and, importantly, were shielded by lipid‐based cloaks that effectively protected them from neutralizing serum while retaining full functionality in cell transduction.^310^

Despite these advancements, challenges in nonviral delivery approaches remain significant and cannot be overlooked. Enhancing the stability and increasing the circulation time of these medicines within the body are critical to ensuring treatment efficacy. Neutral auxiliary lipids, such as dioleoylphosphatidylethanolamine and cholesterol, can improve the colloidal stability of cationic LNPs.^311^ Additionally, PEGylation modifications can reduce nanoparticle aggregation and enhance their stability, thereby improving immune evasion from the reticuloendothelial system.^312^ Furthermore, these vectors must navigate multiple biological barriers, including the endothelium, the extracellular matrix of skeletal muscles, and the cell membrane.^313^ By precisely regulating the physicochemical properties of nonviral vectors—such as surface potential, shape, size, and hardness—the internalization efficiency of cells toward these vectors can be influenced.^314^ Concurrently, the incorporation of cell‐specific ligands on the surface of these vectors can significantly enhance their binding to cell surfaces, a topic that will be explored in a subsequent section.^315^ Moreover, there are intracellular barriers that nonviral vector‐delivered cargos must overcome, including lysosomal escape, intracellular dissociation, and appropriate localization. Selecting an appropriate vector and modifying it with reducible sensitive groups, such as disulfide bonds, may facilitate lysosomal escape and the intracellular release of cargo.^316^ Additionally, cargo linked with nuclear localization sequences (NLS), or covalently modified with NLS peptides and other molecules, can enhance nuclear localization and therapeutic efficacy.^253^

### 5.4 Immunotoxicity of the cargos

The Cas protein‐specific immune response is a significant concern in gene therapy, primarily due to the bacterial and archaeal origins of Cas proteins.^317^ Research has shown that the introduction of SaCas9 into the liver elicits a robust memory T cell response in mice, leading to the near‐complete elimination of gene‐edited hepatocytes.^318^ Similar immune responses have been observed in a dog model of DMD, where gene editing was performed through Cas9‐mediated exon skipping. Although initial assessments indicated substantial dystrophin restoration, the treatment also resulted in muscle inflammation accompanied by Cas9‐specific humoral and cytotoxic T‐lymphocyte responses, despite the muscle‐specific expression of the Cas9 protein.^319^ Consequently, the combination of immunosuppressive therapy during CRISPR/Cas‐based gene editing, such as the introduction of corticosteroids or Cas9‐reactive regulatory T cells, may be considered in clinical practice.^320^ Enhancements to the CRISPR/Cas system represent another potential strategy. Optimized forms of Cas9 proteins with reduced immunogenic epitopes have been developed.^321^ However, further evidence regarding antibody responses to these highly variable epitopes is necessary. Additionally, self‐deleting CRISPR/Cas systems, which utilize sgRNA that targets themselves, along with more specific and efficient editing tools, may further mitigate the immune response associated with CRISPR/Cas‐based gene therapy.^322^

In addition to the foreign protein, it has long been established that microdystrophin can also elicit immune responses during gene replacement therapy, as evidenced by the detection of dystrophin‐specific T cells.^323^, ^324^ Ongoing analyses across several studies suggest that patients with N‐terminal deletions of the dystrophin gene, particularly in regions corresponding to sequences represented in the microdystrophin, may exhibit immune naivete or cross‐reactive immunologic material negativity for these N‐terminal epitopes. This implies that the potentially harmful T‐cell response targeting specific peptides is likely encoded by exons 8–11.^324^ Redesigning the corresponding constructs of microdystrophin to be less likely to provoke an adaptive immune response may offer a viable solution. Alternatively, the codon‐optimized miniaturized utrophin, which serves as the autologous homologue of dystrophin, has been reported to prevent the muscular dystrophy phenotype in mouse and dog models.^325^ Further comparison between microutrophin and microdystrophin, when directly injected into the muscle of a dog model, revealed that, in contrast to the strong T‐cell response observed against microdystrophin, no evidence of cell‐mediated immunity was detected in the microutrophin injected model.^326^

### 5.5 Tissue specifical delivery

Given the toxicity concerns associated with high‐dose systemic delivery of viral vectors, various strategies have been evaluated for their potential to enhance delivery specificity, thereby reducing the necessary viral dosage and improving therapeutic efficacy. The fusion of membranes, directly mediated by fusogens, is a critical step that enveloped viruses utilize to facilitate entry into new hosts.^327^ Additionally, the cell‐cell fusion process occurs during skeletal muscle development and regeneration, with Myomaker and Myomerger acting as fusogens to regulate the fusion of progenitor cells into multinucleated myofibers.^328^ It is particularly promising that muscle cell fusogens could potentially guide gene therapy vectors, significantly enhancing muscle cell delivery specificity. This concept was supported by a recent study demonstrating that the local and systemic injection of an engineered lentivirus, incorporating Myomaker and Myomerger on its membrane, resulted in specific transduction of skeletal muscle. This system exhibited both the capability and specificity for delivering mDystrophin to skeletal muscle in a DMD mouse model, thereby alleviating pathology.^250^ This reported strategy serves as an impressive example, encouraging further research into the development of novel receptor‐binding ligands or protein‐based adaptor molecules, such as Designed Ankyrin Repeat Proteins (DARPin), nanobodies, or single‐chain antibodies, to restrict cell transduction toward target cells in various delivery vehicles, including nonenveloped viral and nonviral vectors.^315^ For instance, a class of AAV capsids containing an arginine‐glycine‐aspartic acid (RGD) motif, as well as a myotropic peptide‐fused capsid, has demonstrated superior efficiency and selectivity for muscle transduction following intravenous injection in both mice and nonhuman primates.^251^, ^329^

Another concern regarding muscle fiber cell‐targeting gene therapy is that the therapeutic effects may be diminished due to transgene loss, which can occur alongside ongoing muscle fiber degeneration and regeneration, as well as during muscle growth in treated children.^330^ Since satellite cells, also known as skeletal muscle stem cells, play a crucial role in the formation of primary and secondary myofibers during muscle development and injury repair,^331^ targeting these cells with gene therapy holds the potential to provide a renewable pool of “corrected” cells for existing muscle fibers. A study utilizing a novel dual reporter mouse model has demonstrated that AAV9 and AAV8 are suitable for transducing satellite cells through both local and systemic injections. Furthermore, the AAV‐delivered Cas9‐based gene editing system possesses the capability to edit satellite cells using muscle‐specific promoters, including CK8e, SPc5‐12, and MHCK7.^332^ These findings underscore the promise of gene therapy, particularly gene editing directed at satellite cells; however, further investigation into delivery methods and expression specificity in large animal models before human trials is necessary.

Conversely, enhancing the cellular or subcellular targeting abilities of the delivered cargos may also improve the tissue specificity and therapeutic efficiency of gene therapy. The myonuclear propagation capability of these cargos can augment overall editing efficiency, as the pathogenic products from nontransduced nuclei in multinucleated muscle cells may dilute the transgenic products.^333^ By screening various combinations of NLS, nuclear export signals, and other elements, a set of short peptide sequences termed “Myospreader” was identified. This sequence promotes Cas9 myonuclear propagation across myofibers, enhances protein stability, and increases muscle gene editing efficiency in vivo.^253^ This underscores the significance of considering the spatial dimension in gene regulation, editing, and therapy.

The key to addressing the muscle‐related issues associated with Pompe disease lies in the effective delivery of glucosylceramidase to muscle tissue. To enhance the targeting of glucosylceramidase to muscles, a HSC‐mediated lentiviral gene therapy was developed, which expresses an insulin‐like growth factor 2 (IGF2) peptide fused to glucosylceramidase (designated LV‐IGF2.GAAco). This approach is based on the principle that ex vivo gene‐modified autologous HSPCs can stably overexpress the therapeutic transgene, while the IGF2 peptide increases binding affinity to the cation‐independent mannose‐6‐phosphate/IGF2 receptor (M6PR/IGF2R).^255^, ^334^ Preclinical data demonstrated nearly complete phenotypic and proteomic correction in the primary affected organs of a mouse model when compared with LV‐GAA alone, indicating its potential for further clinical applications.^255^, ^256^

### 5.6 Tissue specifical expression

Successful gene therapy depends not only on the selection of an appropriate vector but also on the specific expression of the appropriate exogenous genes in targeted tissues. A properly selected promoter for transgene expression can facilitate long‐term, sustained high expression in muscle tissues while demonstrating limited activity in other areas. This is crucial for the gene therapy of muscular disorders. Specifically, a short regulatory sequence that effectively drives high and robust expression of a therapeutic transgene in various muscle types, while exhibiting minimal activity in nontarget tissues, is vital for multiple gene therapy strategies addressing genetic muscular disorders.

An earlier review summarized the commonly used muscle‐specific promoters based on skeletal muscle α‐actin, MCK, and desmin genes, and provided information on current gene therapy candidates that utilize these promoters.^335^ A more recent study compared some frequently used muscle‐specific promoters and demonstrated that the Desmin and MHCK7 promoters exhibited stronger reporter gene expression levels in myogenic cell lines and also promoted gene expression in cardiac cells, whereas miR206 and CAPN3 promoter expression was restricted to skeletal muscle.^336^ Despite advancements in the understanding of these natural promoters, there remains a demand for higher therapeutic gene expression levels under specific treatment conditions. The construction of a novel muscle hybrid (MH) promoter may address these additional requirements. Research data indicated that when combined with AAV2/9, the MH promoter—composed of the Des gene enhancer, Ckm gene enhancer, modified Ckm gene core promoter, and a small intronic enhancer derived from the Ckm gene—demonstrated higher in vivo expression levels in skeletal muscle and the heart compared with CMV, desmin, or CKM‐based promoters, regardless of whether delivery was intramuscular or systemic.^337^ Furthermore, a multistep computational approach employing a genome‐wide data‐mining strategy has been reported to design robust muscle‐specific transcriptional cis‐regulatory modules, resulting in a substantial increase in muscle‐specific gene transcription (up to 400‐fold) when delivered with AAV in mice, without any discernible immune complications.^338^

### 5.7 CNS delivery

Several types of genetic muscular disorders, including DMD and Pompe disease, are associated with CNS involvement.^19^, ^20^ However, the ability of gene therapies to traverse the blood–brain barrier is limited when delivered systemically. To address this limitation, numerous studies have proposed IT delivery methods, which target the CNS and peripheral organs via cerebrospinal fluid, significantly enhancing cargo delivery to the brain and spinal cord. In two studies involving DMD mouse models, AONs delivered via ICV and intra‐cisterna magna routes demonstrated detectable exon 51 skipping in various brain regions. This was linked to partial improvements in anxiety traits, unconditioned fear responses, and Pavlovian fear learning and memory.^247^, ^248^ Regarding viral‐based delivery, a single IT administration of AAV–hGAA in 1‐month‐old GAA–KO 6neo/6neo mice was performed, resulted in reduced glycogen levels in the brainstem, spinal cord, and left cardiac ventricular wall. Neurological correction, neuromuscular improvement, and the resolution of hypertrophic cardiomyopathy were monitored at 4, 9, and 12 months posttreatment.^339^

### 5.8 Off‐target issues

Beyond the immunogenicity of the vector and Cas protein discussed, the potential for off‐target activity in CRISPR/Cas systems also presents a barrier to their clinical application. While most off‐target cleavage events have been reported in highly proliferating cells in culture, they were believed to be minimal in postmitotic skeletal and cardiac cells.^340^ However, the unexpectedly high rate and diverse distribution of AAV gene insertion into the host genome via CRISPR‐induced DSBs during gene therapy in muscle cells raises concerns about off‐target effects in therapies for muscular disorders.^222^ Consequently, the risk of deleterious off‐target mutations in susceptible cells in vivo cannot be disregarded, particularly with systemic administration. Various strategies have been developed to mitigate off‐target mutagenesis, including the careful selection and design of sgRNAs, the development and use of high‐fidelity Cas enzymes, and the implementation of less mutagenic tools, alongside the application of muscle cell‐specific vectors and promoters as discussed above. Furthermore, the delivery of RNPs containing recombinant Cas9 protein and guide RNA, rather than transgenes, has demonstrated immediate cleavage while reducing off‐target sites across multiple cell types due to the rapid degradation of these particles within cells.^341^ This approach offers another potential delivery method in conjunction with nonviral vectors for gene editing.

In conjunction with traditional biochemical methods, the enriched databases related to gene editing and the advancements in artificial intelligence technology over the past few years have led to the emergence of data‐driven machine learning methods as a powerful new approach for predicting both on‐target and off‐target effects of the CRISPR/Cas system.^342^ Currently, both traditional machine learning models and deep learning models are being utilized in this field. Numerous tools have been developed using traditional machine learning models to predict off‐target sites, design optimized sgRNA libraries that maximize on‐target activity while minimizing off‐target effects,^343^ and extract gRNA knockdown efficiency based on specific sequence features.^344^ As gene editing datasets continue to expand, deep learning models have demonstrated significant advantages in processing large volumes of data characterized by complex nonlinear patterns. Reports indicate that deep learning models yield superior prediction results, as measured by AUPRC and AUCROC, when employing novel sgRNA–DNA sequence encoding strategies compared with traditional models.^345^ Furthermore, biological features beyond sequence patterns can also be incorporated into deep learning models for predictive purposes. For instance, the binding energy estimations of the gRNA‐DNA hybrid as well as the Cas9–gRNA–DNA hybrid have been successfully utilized to predict on‐target activity and off‐target sites.^346^ Additionally, deep learning models have been applied to address data imbalance issues that hinder off‐target prediction,^347^ as well as to enhance feature explainability for accurate on‐ and off‐target predictions,^348^ underscoring their emerging importance in predicting gene editing effects.

## 6 CONCLUSIONS

Over the past decade, gene therapy has emerged as a promising alternative, offering the potential for one‐time treatments for diseases and significantly influencing the management of rare disorders. Approaches such as gene replacement and gene editing have shown significant advantages in both clinical and preclinical studies, owing to their vast potential through various strategies. Nevertheless, in comparison with small molecule therapies, antibody therapies, and protein replacement therapies, gene therapy remains in the early stages of development, with many challenges yet to be addressed.

The field of gene therapy for genetic diseases must address several critical issues, including treatment persistence, safety, and accessibility. Many clinical trials have only recently begun (see Table 2), and a significant number of these trials have yet to publish results. Additionally, numerous studies remain in the preclinical investigation phase. The integration of gene therapy with other modalities, such as cell therapy, represents a promising strategy to mitigate immune complications in vivo while ensuring sustained therapeutic effects at targeted sites. However, a comprehensive evaluation of the delivery and therapeutic efficiency of engineered cells in vivo is essential before clinical applications can proceed. Consequently, long‐term follow‐up is crucial for establishing the durability of gene therapy. Regarding safety, the immunogenicity of AAV and cargo proteins, along with the presence of pre‐existing antibodies against them in patients, present challenges to the clinical application of gene therapy. Moreover, safety concerns arising from off‐target effects associated with gene editing technologies must also be addressed. Additionally, the accessibility of these therapeutic approaches should be considered in clinical practice. Cost‐effectiveness is an important factor, as gene therapy applications are often very expensive interventions. The marginal clinical effects of existing strategies, as well as the cost differences among various developing gene therapy methods, may also significantly influence the clinical application of these therapies.

As a pivotal technology in gene therapy, advancements in delivery mechanisms are poised to significantly broaden the application scope of this therapeutic approach. The development of tissue‐specific, efficient, safe, and low‐immunogenicity gene therapy vectors necessitates ongoing optimization due to the packaging limitations and toxicity associated with high doses of AAV observed in several current trials. Various directed evolution and rational design modifications are employed on AAV to facilitate safe and effective delivery to targeted tissues while minimizing distribution to nontarget organs. Additionally, machine learning models present a promising strategy for engineering novel AAV capsids, leveraging the accumulation of data from long‐term research on viruses and the immune system. Furthermore, the development of more effective nonviral vectors, such as LNPs, viroids, and EVs, is expected to contribute to the reduction of production costs. Beyond vector evolution, the incorporation of innovative tissue‐specific promoters and subcellular localization subunits, as well as sequence optimization for the cargos themselves, can facilitate the achievement of similar therapeutic effects while reducing the required dosage and immunogenicity. Future research must also address the limitations in our understanding of the biological mechanisms associated with a broader range of genetic disorders, which currently constrains the expansion of gene therapy applications. Strengthening our biological understanding of these diseases is imperative, and this can be supported through the development of reliable novel animal models and ex vivo models derived from clinical samples. For instance, mouse models exhibiting Cre‐mediated skeletal muscle fiber‐specific Cas9 expression present a promising method for screening different disease states and identifying candidate genes,^349^ while three‐dimensional organotypic skeletal muscle cultures derived from patient‐iPSCs offer a practical model for studying muscle development, maturation, disease, and repair in vitro.^350^, ^351^, ^352^, ^353^

In summary, future endeavors in gene therapy for genetic disorders should prioritize the expansion of treatment options and modalities, the attainment of targeted delivery to specific tissues and organs, and the enhancement of long‐term efficacy and safety. These advancements have the potential to significantly improve patient survival and quality of life in a personalized manner.

## AUTHOR CONTRIBUTIONS

Beibei Qie and Jianghua Tuo performed the literature search and wrote the manuscript. Feilong Chen, Haili Ding and Lei Lyu revised the manuscript. Lei Lyu conceived and supervised the review and wrote and revised the manuscript. All the authors have read and approved the final version of the manuscript.

## CONFLICT OF INTEREST STATEMENT

The authors declare no conflicts of interest.

## ETHICS STATEMENT

Not applicable.

## Data Availability

Not applicable.
